# Prevalence and factors associated with mental illness symptoms among school students post lockdown of the COVID-19 pandemic in the United Arab Emirates: A cross-sectional national study

**DOI:** 10.1371/journal.pone.0296479

**Published:** 2024-02-01

**Authors:** Nariman Ghader, Noor AlMheiri, Asma Fikri, Hira AbdulRazzak, Hassan Saleheen, Basema Saddik, Yousef Aljawarneh, Heyam Dalky, Ammar Al Banna, Shammah Al Memari, Budoor Al Shehhi, Shereena Al Mazrouei, Omniyat Al Hajeri

**Affiliations:** 1 Mental Health Department, Emirates Health Services, Dubai, United Arab Emirates; 2 National Center for Health Research, Ministry of Health and Prevention, Dubai, United Arab Emirates; 3 Statistics and Research Center, Ministry of Health and Prevention, Dubai, United Arab Emirates; 4 Abu Dhabi Public Health Center, Department of Health, Abu Dhabi, United Arab Emirates; 5 Department of Family and Community Medicine, University of Sharjah, Sharjah, United Arab Emirates; 6 Faculty of Health Sciences, Higher Colleges of Technology, Dubai, United Arab Emirates; 7 Child and Adolescent Mental Health Center of Excellence, Al Jalila Children’s Specialty Hospital, Dubai, United Arab Emirates; McMaster University, CANADA

## Abstract

Limited data exists on the mental health of children in the United Arab Emirates (UAE). This study aimed to fill this gap by examining the prevalence of anxiety, depression, and risk for Post-Traumatic Stress Disorder (PTSD) among school students in post-lockdown of the COVID-19 pandemic. A sample of 3,745 school students participated, responding to standardized tests (Mood and Feeling Questionnaire-Child Self-Report, Screen for Child Anxiety Related Disorders-Child Version, and Children’s Revised Impact of Event Scale-8). Findings showed that the risk for PTSD was the most prevalent (40.6%), followed by symptoms of anxiety (23.3%), and depression (17.1%). For gender differences, symptoms of the three conditions (depression, anxiety, and PTSD) were higher in female students (9.2%) compared to male peers (7.7%) (p = 0.09). Moreover, symptoms of depression and anxiety were found to be higher among late adolescents (p<0.05). Further analysis revealed that having medical problems was a positive predictor for anxiety (OR = 2.0, p<0.01) and risk for PTSD (OR = 1.3, p = 0.002); similarly, witnessing the death of a close family member due to COVID-19 (OR for depression, anxiety, and PTSD = 1.7, p<0.01) were positive predictors associated with PTDS, depression, and anxiety. The study concluded that post COVID-19 lockdown, symptoms of anxiety, depression, and risk for PTSD were found to be prevalent among school students in the UAE. Researchers put forward recommendations on the initiation of a national school mental health screening program, the provision of follow-up services for vulnerable students, and the integration of a mental health support system in the disaster preparedness plans.

## Introduction

The outbreak of the COVID-19 pandemic in early 2020 triggered an unprecedented global health crisis, resulting in substantial morbidity, mortality, and pervasive disruptions to daily life. As a response to mitigate the spread of the virus, stringent public health measures, including nationwide lockdowns were implemented across many countries, including the United Arab Emirates (UAE). These measures aimed to curb the transmission of the virus; however, they inadvertently imposed significant psychological and social challenges, particularly among school students. Over the course of this period, mental health has emerged as a critical focal point, garnering significant attention. The World Health Organization (WHO) confirmed that a substantial proportion of nations, approximately 90%, encountered substantial disruptions in the basic healthcare services, coinciding with a marked surge in the requisition for mental health services [1].

Additionally, the Policy Brief on the need for action on mental health released by the United Nations (UN) in 2020 affirmed that the COVID-19 crisis has affected the mental health and overall well-being of entire communities, necessitating immediate attention and prioritization [2]. While, focusing on mitigating the distinct impacts of COVID-19 on children, the UN Policy Brief concerning the influence of COVID-19 on children emphasized the detrimental effects of measures such as physical distancing and movement restrictions on children’s mental well-being. It also underscored the increased vulnerability of anxiety within this demographic [3]. Globally, the prevalence of depression, stress, and anxiety among the general population during the COVID-19 pandemic is estimated to be 33.7%, 29.6%, and 31.9% respectively [4]. The escalating risk of contracting the infection, harrowing narratives of pain and mortality, enforced measures and regulations (including social isolation, lockdowns, and vaccinations), the pervasive information frenzy in the media, and the ensuing economic ramifications have collectively engendered a global atmosphere of heightened depression and anxiety [5]. Studies have revealed an increased risk of post-traumatic stress disorder (PTSD), sleep disorders, and cognitive underperformance in comparison to the pre-pandemic period [6].

Conventionally, children and young people are at the heart of global development. However, in a press release by UNICEF in October 2021, it was affirmed that the younger generation has been carrying the burden of mental health conditions even prior to COVID-19 [7]. According to the State of the World’s Children 2021 report, one in seven adolescents aged 10–19 years is estimated to suffer from a mental disorder; additionally, the report warned that children and young people may experience the impact of COVID-19 on their mental health and well-being for many years to come [8]. In a systematic review on the mental health of children and young people before and during COVID-19 pandemic, data from 21 studies across 11 countries showed that since the pandemic has started there was a longitudinal decline in the overall mental health status of adolescents and young people with preponderance of depression, anxiety and psychological distress [9]. Further evidence showed that uncertainties associated with the pandemic itself, such as prolonged and widespread parental stressors, school closures, and loss of loved ones have been detrimental to the well-being of adolescents and children [10].

In the pre-pandemic era, a comprehensive review of relevant global and national initiatives addressing mental health challenges reveals a dynamic landscape shaped by concerted efforts to promote mental well-being, alleviate stigma, and enhance access to mental health services. On a global scale, initiatives such as the World Health Organization’s Mental Health Action Plan emphasize the integration of mental health into overall health policies and the development of community-based mental health services [11]. Additionally, international collaborations, such as the Global Mental Health Movement, underscore the need for a collective response to address the multifaceted nature of mental health issues [12]. In the United Arab Emirates (UAE), mental health was recognized as a national priority which resulted in the release of the 2017 National Policy for Mental Health Promotion by the Ministry of Health and Prevention [13]. Despite this focus, a national report by Janssen indicated that mental disorders, particularly depression and anxiety, accounted for a substantial portion (19.9%) of the UAE’s disease burden [14]. In response to this issue, the UAE strategically initiated mental health initiatives, including the launch of the National Campaign for Mental Support, led by the National Program for Happiness and Wellbeing, during the pandemic [15].

Nevertheless, consistent with the global trend, local studies demonstrated a noticeable mental health impact within the population. For instance, a study focusing on the psychological well-being of university students in the UAE during the pandemic revealed that over 50% of students reported anxiety levels ranging from mild to severe, with higher levels reported by females [16]. Additionally, a separate study conducted in the UAE showcased increased levels of anxiety and depression in the adult community compared to pre-pandemic figures. Furthermore, this study highlighted significant associations between mental symptoms (depression and anxiety) and factors such as youth, gender (female), prior history of mental health issues, and personal or loved ones’ positive COVID-19 test results [17].

Indisputably, studies on the mental health of the UAE’s younger population are essential, given that 17–22% of the nation’s youth suffer from depressive symptoms [18]. These findings underscore the critical need to address mental health issues among the youth, emphasizing the urgency for targeted interventions and support measures to alleviate mental distress in this demographic. Expectedly, the emergence of COVID-19 has exacerbated this state of disturbance. It has been acknowledged that during the pandemic, children in the UAE were facing increased mental health issues associated with stories of calamities, home confinement, and fear of contracting the virus [19]. In a UAE study that explored the anxiety levels among adults and children during the COVID-19 pandemic, findings showed that the overall prevalence of Generalized Anxiety Disorder was 71% in the total population and 59.8%) in the younger generation [20]. Moreover, a study focusing on policy development for children’s mental health during the COVID-19 pandemic anticipated a rise in the volume of individuals seeking mental health assistance [21].

Understanding the mental health impact of the pandemic and the subsequent lockdown measures on school students is of paramount importance. Identifying the prevalence of mental illness symptoms and associated factors in this population post-lockdown is crucial for developing targeted interventions, providing appropriate mental health support, and informing future public health strategies. Given the limited specialized research on children mental health before and during the COVID-19 pandemic in the UAE, this study aimed to establish a comprehensive data reference regarding the prevalence of mental illness symptoms (depression, anxiety, and PTSD) and to determine the associations of these symptoms with bio-demographic and other pandemic-related factors among UAE school students. The study’s outcomes will serve as valuable scientific evidence, shedding light on the scale and characteristics of required efforts in children’s mental health.

## Methodology

### Study design

A descriptive cross-sectional study was conducted between February 2021 and July 2021 to establish a comprehensive data reference regarding the prevalence of mental illness symptoms (depression, anxiety, and PTSD) and to determine the associations of these symptoms with bio-demographic and other pandemic-related factors among UAE school students. This design was selected as lit has been affirmed by literature as the best way to describe the distribution of one or more variables, devoid of causal links. Furthermore, this type of studies has been considered instrumental in measuring the prevalence of a disease or of a risk factor in a population [22].

### Sampling and data collection procedure

In this cross-sectional study, data was gathered through online means between February 2021 and July 2021 in the UAE. This timeframe followed a period of lockdown, during which a majority (83.3%) of students were still engaged in complete online school learning. A total of 3,745 respondents from all seven emirates of the United Arab Emirates participated in the study. Participation was open to students from both private and government schools in the UAE, encompassing grades 2 to 12 and spanning ages 8 to 18. The study targeted individuals with the ability to competently comprehend written Arabic and/or English texts. Those enrolled in vocational, continuing education, or special education programs/schools were excluded. Furthermore, parents of surveyed students responded to a series of biodata inquiries through the same instrument.

Our study utilized convenience and snowball sampling techniques, which have been widely used in similar research conducted amidst the COVID-19 pandemic [23–25]. This approach was chosen due to the logistical challenges and restrictions posed by the pandemic, making it a practical method for data collection under the circumstances. Initially, potential participants were reached electronically through school and parental communication channels. Additionally, the study harnessed electronic communication platforms associated with the authors’ corporate affiliations. This approach involved disseminating the study invitation and link to the public through official websites, press releases, and various social media outlets such as Facebook, Instagram, and Twitter. Moreover, to ensure comprehensive outreach, the study utilized the established networks of corporate facilities, including hospitals and clinics, to circulate the invitation via SMS to the employees’ families who met the inclusion criteria. This innovative dissemination technique facilitated wider access to the study’s invitation.

### The instrument

The data collection instrument used in this study was composed of three sections. Firstly, a detailed participant information sheet and an informed consent form were mandatory fields to be completed before participants had access to the anonymous questionnaire. Secondly, parents were asked to respond to 26 questions which included family sociodemographic data as well as information on the student’s general health status; the estimated time to complete this part was 15 minutes. Thirdly, students, just after they consented to their participation, were requested to answer blocks of questions that included three standardized scales tested for validity and reliability in both languages (English and Arabic).

The Mood and Feelings Questionnaire (Short Version)–MFQ is a self-reported 13-item measure that assesses the current depressive symptomatology among children. Psychometric data at an early stage have revealed a high internal consistency along with a Cronbach’s alpha of 0.90. Test-retest reliability across the clinical samples for a 1-week period is wide-ranging between 0.73 and 0.75 (intraclass correlation) [26]. The “Screen for Child Anxiety Related Emotional Disorders (SCARED)” is a youth- and parent-report measure that was established to screen children for anxiety related disorders. SCARED comprises of 41 items. In prior investigations involving outpatient psychiatric samples, the SCARED demonstrated favorable convergent and divergent validity when compared to other psychiatric assessment tools, indicating robust sensitivity (0.71) / specificity (0.67) [27, 28]. The “Children’s Revised Impact of Events Scale” (CRIES-8) realizes the principles for good screening instruments and has been utilized across a large number of cultures and countries. CRIES has exhibited strong reliability, a consistent factor structure, satisfactory face and construct validity, and has been employed to screen extensive cohorts of vulnerable children following various traumatic events [29]. The cutoff values for these scales were established based on previous research) [26–32].

### Data quality assurance

To ensure the accuracy and reliability of the data collected for this study, stringent measures were implemented throughout the research process. These procedures were designed to tackle potential issues related to completeness, accuracy, and the handling of unanswered responses. A standardized data collection protocol was developed, clearly outlining the procedures for obtaining responses from participants. Although the questionnaire utilized standardized scales to ensure reliability and validity, a pilot test was conducted as an additional measure. This preliminary trial involved a select group of individuals mirroring the study’s target participants. Its purpose was to detect any potential shortcomings, enhance clarity, and optimize user experience, ultimately refining the questionnaire’s effectiveness for the larger study. Feedback from the pilot test guided adjustments to ensure the questionnaire was user-friendly and the instructions provided proper guidance. After the completion of data collection, thorough data cleaning procedures were applied. The percentage of unanswered responses for each variable was also reported for transparency purposes. These data quality assurance measures collectively aimed to enhance the reliability and inclusiveness of the dataset, ensuring the robustness of the study findings [33].

### Privacy and confidentiality

Data were collected anonymously and were confidentially stored in the Emirates Health Services database system. Investigators and statisticians who helped in data analysis were the only individuals authorized to access the data and only after signing a non-disclosure agreement form. Moreover, to facilitate transparency and replication of the study’s findings, an anonymized dataset essential for reproducing the study’s results has been made accessible to fellow researchers. This dataset is publicly available on a designated repository [34], promoting collaboration and the advancement of knowledge in the field while upholding the confidentiality of participant information.

### Ethical considerations

Ethical approvals were acquired from the Emirates Institutional Review Board for COVID-19 Research (Reference No. DOH/CVDC/2020/2471) and the Research Ethics Committee at the Ministry of Health and Prevention, Dubai (reference No. MOHAP/DXB-REC/ DDD/No.163 /2020). Moreover, anonymity, confidentiality, and voluntariness were preserved throughout the study. As is conventional in anonymous surveys, consent was in place before starting the survey. Parents confirmed their consent in the electronic questionnaire by answering two written mandatory questions placed just at the end of the Participant Information Sheet. Students were also requested to confirm their consent by responding to one mandatory written question just before they answered the electronic survey questions. Participants were informed they could stop completing the questionnaire or refrain from submitting their responses if they felt uncomfortable at any stage during completion. Participation in the study was voluntary. Participants had the full right to withdraw at any time without the need to justify their actions. There were no apparent risks that could result from participating in this study. However, due to the fact that some students could feel uncomfortable expressing their feelings, a mental health support hotline was made accessible to participants in case they needed relevant professional consultations.

### Data analysis

Data were analyzed using IBM SPSS Statistics for Windows version 26. The number of submitted questionnaires was 3,762. However, post-data cleaning, the number was reduced to 3,745. A combination of descriptive and inferential statistical techniques were implemented to achieve the study goal. Descriptive statistics were used to present the distribution of students across different age groups, gender, and various conditions related to the current coronavirus pandemic. The inferential statistics included chi-squared tests (χ^2^) and p-values to assess the association between categorical variables (age groups, gender, and the presence of symptoms) and to analyze the relationship between various factors (student medical problems, method of school learning, coronavirus-related experiences of the student and their family members) and the presence of symptoms (Depression, Anxiety, PTSD). Moreover, logistic regression analysis with Odds Ratios (OR) along with 95% confidence intervals (CIs) and p-values were used to assess the association between different categorical variables and the presence of symptoms (Depression, Anxiety, PTSD). A P-value < 0.05 was considered statistically significant in all analyses. In constructing our multivariable logistic regression model, we meticulously navigated the variable selection process by considering a range of factors to ensure both statistical robustness and theoretical relevance to our research question. The prioritization of variables was grounded in a theoretical assessment of their relevance to the research objectives, with a focus on those directly linked to the investigated phenomenon. Our approach was further informed by a comprehensive literature review, incorporating insights from prior studies and established theoretical frameworks. To bolster the clinical validity of our model, we sought input from domain experts, including clinicians, to align selected variables with relevant clinical knowledge. Additionally, we employed statistical tests, such as chi-square tests or others depending on variable nature, to evaluate the significance of variables.

This comprehensive statistical approach allowed for a nuanced exploration of the factors influencing mental health symptoms among students in the UAE, providing valuable insights into the interplay between various variables and mental well-being.

### Findings

The socio-demographic characteristics of participants shown in Table 1 were derived from the section answered by the parents. The majority of parents were married (94.2%), employed (81.4%), and expats (67.8%). Likewise, most respondents (93.5%) had none of their family members been previously diagnosed with a mental health-related problem or behavioral disorder. As for students, over one-third (34.3%) were pre-adolescents (<10 years), 37.5% belonged to the early adolescence age group (10–13 years), 23.7% to middle adolescence (14–17 years), and 1.8% to late adolescence (22 years). It is to be noted that these age groups were defined based on the UAE stages of school education [35] and a published article on stages of adolescence [36]. For gender, a little over half (50.6%) of the participants were male. Most of the students (81.6%) studied in private schools and attended school completely online (82.3%).

**Table 1:** Socio-demographic characteristics of the sample (N = 3745).

Variables	Number (%)*
**Person responding to this questionnaire**	
Father	1227 (32.8)
Mother	2120 (56.6)
Both parents/ Legally Authorized Representative/Guardian	398 (10.6)
Total	3745 (100)
**Parents Marital status**	
Married	3528 (94.2)
Divorced	125 (3.3)
Separated	53 (1.4)
Widowed	39 (1.0)
Total	3745 (100)
**Total number of people living in house at present**	
2–4	1442 (38.5)
5–7	1719 (45.9)
8–10	358 (9.6)
More than 10	219 (5.8)
Not answered or not applicable (N/A)	7 (0.2)
Total	3738 (100)
**Nationality**	
Emirati	1201 (32.1)
Non-Emirati	2540 (67.8)
N/A	4 (0.1)
Total	3741 (100)
**Employment status (parents/guardian/legally authorized representatives)**	
Employed	3051 (81.4)
Unemployed	694 (18.5)
Total	3745 (100)
**Family member diagnosed with a mental health-related problem or behavioral disorder**	
Yes	231 (6.2)
No	3503 (93.5)
N/A	11 (0.3)
Total	3734 (100)
**Age of student (years) **	
Pre-adolescence (<10 years)	1285 (34.3)
Early adolescence (10–13 years)	1403 (37.5)
Middle adolescence (14–17 years)	888 (23.7)
Late adolescence (18 years)	67(1.8)
N/A	102 (2.8)
Total	3643 (100)
**Gender of student **	
Male	1895 (50.6)
Female	1800 (48.1)
N/A	50 (1.33)
Total	3695 (100)
**Student has any medical problems**	
Yes	259 (6.9)
No	3486 (93.1)
Total	3745 (100)
**Student being diagnosed with a mental health-related problem or behavioral disorder**	
Yes	114 (3.0)
No	3631 (97.0)
Total	3745 (100)
**Student school category**	
Private	3057 (81.6)
Governmental	517 (13.8)
Semi-governmental	137 (3.7)
N/A	34 (0.9)
Total	3711 (100)
**Student current method of school learning**	
Completely online	3083 (82.3)
Hybrid (partly online)	520 (13.9)
Completely live at school	109 (2.9)
N/A	33 (0.9)
Total	3712 (100)

In Table 2, data were also collected from parents’ questions. It showed that 14% had a close family member at the time tested positive for coronavirus, followed by 14% who indicated that a close family member was before or at the time sick/hospitalized because of the current coronavirus infection. However, only 5.8% reported that a close family member had died because of the coronavirus. A smaller percentage of students (3%) tested positive for coronavirus at the time while 5.4% were either unwell or hospitalized.

**Table 2:** COVID-19 information pertaining to the sample (N = 3,745).

Variables	Number (%)
**A close family member has currently tested positive for coronavirus**	
True	532 (14.2)
False	2998 (80.1)
Not sure	215 (5.7)
**A close family member is/got sick or is/was hospitalized because of the current coronavirus infection**	
True	525 (14.0)
False	3072 (82.0)
Not sure	148 (4.0)
**You work around people who might have the current coronavirus**	
True	798 (21.3)
False	2291 (61.2)
Not sure	656 (17.5)
**A close family member died because of the current coronavirus**	
True	216 (5.8)
False	3367 (89.9)
Not sure	162 (4.3)
**Student is currently tested positive for coronavirus**	
True	113 (3.0)
False	3516 (93.9)
Not sure	116 (3.1)
**Student is/got sick or is/was hospitalized because of the current coronavirus pandemic**	
True	203 (5.4)
False	3419 (91.3)
Not sure	123 (3.3)

**Fig 1:**
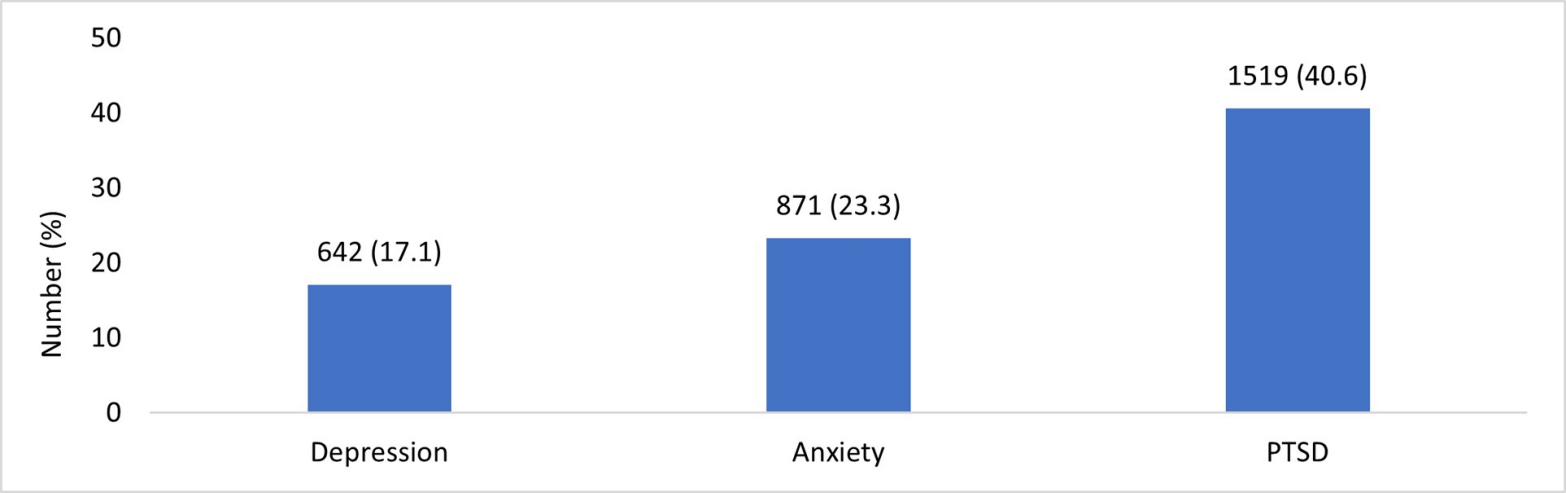
Prevalence of common mental health disorders.

A comprehensive examination of the outcome variables, which encompassed mental health symptoms as self-reported by the students, revealed substantial variability. Among the 3,745 students, 642 (17.1%) reported depression symptoms on the MFQ-Child Self-report scale, which utilized a total score range of 0–26 with a suggested cutoff of ≥12. On SCARED-Child version scale, among the 3,745 students, 871 (23.3%) exceeded the scale’s cutoff (>30), signifying the potential presence of an anxiety disorder. Similarly, symptoms indicative of a risk for PTSD were observed in 1519 (40.6%) students who met or surpassed the defined cutoff score of ≥ 17 on CRIES-8 scale (Fig 1.).

Further inferential analysis looked into trends and associations among the outcome variables and predictive variables, age and gender. The data in Table 3 underscores intriguing patterns and disparities. Significant age and gender differences were found to be associated with mental health symptoms. A significantly higher proportion of participants who were late adolescents reported symptoms of two conditions (depression and anxiety) compared to participants in the younger age group (p<0.05). Additionally, a significantly higher proportion of female students reported symptoms of one condition-depression (p<0.05), anxiety (p<0.01), or risk for PTSD only (p<0.05) or two conditions (p<0.05).

**Table 3:** Distribution of symptoms of depression, anxiety, and risk for PTSD by age and gender.

	Age of student (years)					Gender of student		
	Pre-adolescence (<10 years) [n = 1285]	Early adolescence (10–13 years) [n = 1403]	Middle adolescence (14–17 years) [n = 888]	Late adolescence (18 years) [n = 67]	*p*-value	Male [n = 1895]	Female [n = 1800]	*p*-value
**Symptoms of one condition**								
Depression only	218 (17.0)	246 (17.5)	153 (17.2)	18 (26.9)	0.22	301 (15.9)	333 (18.5)	0.035
Anxiety only	288 (22.4)	328 (23.4)	219 (24.7)	16 (23.9)	0.68	377 (19.9)	482 (26.8)	<0.001
PTSD only	499 (38.8)	593 (42.3)	371 (41.8)	27 (40.3)	0.30	730 (38.5)	765 (42.5)	0.014
**Symptoms of two conditions**								
Depression and anxiety	125 (9.7)	153 (10.9)	114 (12.8)	12 (17.9)	0.016	177 (9.3)	224 (12.4)	<0.01
Depression and PTSD	146 (11.4)	184 (13.1)	104 (11.7)	11 (16.4)	0.35	207 (10.9)	237 (13.2)	0.014
Anxiety and PTSD	198 (15.4)	254 (18.1)	151 (17.0)	12 (17.9)	0.31	279 (14.7)	340 (18.9)	<0.01
**Symptoms of three conditions**								
Depression, anxiety, and PTSD	96 (7.5)	130 (9.3)	81 (9.1)	8 (11.9)	0.24	146 (7.7)	166 (9.2)	0.09

A more detailed examination of the relationship between pandemic-related factors and mental health symptoms is shown in Table 4. Students who were reported to have medical problems were more likely to have mental health symptoms as compared to those with nil history (p<0.01). Those who reported a close family member was sick or hospitalized because of the coronavirus infection were more likely to report mental health symptoms as compared to those who reported the statement was false (p<0.05). In terms of the death of a close family member due to coronavirus infection, respondents who reported a close family member had died because of the coronavirus infection were more likely to show mental health symptoms as compared to those who did not (p<0.01).

**Table 4:** Prevalence of symptoms of depression, anxiety, and PTSD stratified by pandemic-related factors.

	Number (%)	Depression			Anxiety			PTSD		
		Present	Absent	*p-value*	Present	Absent	*p-value*	Present	Absent	*p-value*
**Student has any medical problems**										
Yes	259 (6.9)	68 (26.3)	191 (73.7)	<0.01	107 (41.3)	152 (58.7)	<0.01	131 (50.6)	128 (49.4)	<0.01
No	3486 (93.1)	574 (16.5)	2912 (83.5)		764 (21.9)	2722 (78.1)		1388 (39.8)	2098 (60.2)	
**Student current method of school learning**										
Completely online	3083 (83.1)	511 (16.6)	2572 (83.4)	0.025	734 (23.8)	2349 (76.2)	0.10	1242 (40.3)	1841 (59.7)	0.63
Hybrid (partly online)	520 (14.0)	98 (18.8)	422 (81.2)		102 (19.6)	418 (80.4)		221 (42.5)	299 (57.5)	
Completely live at school	109 (2.9)	28 (25.7)	81 (74.3)		27 (24.8)	82 (75.2)		44 (40.4)	65 (59.6)	
**Student currently tested positive for coronavirus**										
True	113 (3.0)	21 (18.6)	92 (81.4)	0.66	33 (29.2)	80 (70.8)	0.08	53 (46.9)	60 (53.1)	0.38
False	3516 (93.9)	598 (17.0)	2918 (83.0)		804 (22.9)	2712 (77.1)		1419 (40.4)	2097 (59.6)	
Not sure	116 (3.1)	23 (19.8)	93 (80.2)		34 (29.3)	82 (70.7)		47 (40.5)	69 (59.5)	
**Student is/got sick or is/was hospitalized because of the current coronavirus pandemic**										
True	203 (5.4)	46 (22.7)	157 (77.3)	0.09	60 (29.6)	143 (70.4)	0.007	99 (48.8)	104 (51.2)	0.007
False	3419 (91.3)	574 (16.8)	2845 (83.2)		775 (22.7)	2644 (77.3)		1373 (40.2)	2046 (59.8)	
Not sure	123 (3.3)	22 (17.9)	101 (82.1)		36 (29.3)	87 (70.7)		47 (38.2)	76 (61.8)	
**A close family member is currently tested positive for coronavirus**										
True	532 (14.2)	106 (19.9)	426 (80.1)	0.15	139 (26.1)	393 (73.9)	0.002	229 (43.0)	303 (57.0)	0.45
False	2998 (80.1)	503 (16.8)	2495 (83.2)		668 (22.3)	2330 (77.7)		1204 (40.2)	1794 (59.8)	
Not sure	215 (5.7)	33 (15.3)	182 (84.7)		64 (29.8)	151 (70.2)		86 (40.0)	129 (60.0)	
**A close family member is/got sick or is/was hospitalized because of the current coronavirus infection**										
True	525 (14.0)	109 (20.8)	416 (79.2)	0.04	165 (31.4)	360 (68.6)	<0.01	238 (45.3)	287 (54.7)	0.028
False	3072 (82.0)	512 (16.7)	2560 (83.3)		674 (21.9)	2398 (78.1)		1219 (39.7)	1853 (60.3)	
Not sure	148 (4.0)	21 (14.2)	127 (85.8)		32 (21.6)	116 (78.4)		62 (41.9)	86 (58.1)	
**A close family member died because of the current coronavirus**										
True	216 (5.8)	57 (26.4)	159 (73.6)	<0.01	78 (36.1)	138 (63.9)	<0.01	119 (55.1)	97 (44.9)	<0.01
False	3367 (89.9)	553 (16.4)	2814 (83.6)		740 (22.0)	2627 (78.0)		1326 (39.4)	2041 (60.6)	
Not sure	162 (4.3)	32 (19.8)	130 (80.2)		53 (33.7)	109 (67.3)		74 (45.7)	88 (54.3)	

Following a thorough regression analysis, the study substantiates the factors associated with depression, anxiety, and risk for PTSD as shown in Table 5. Notably, students who had any medical problems demonstrated a 2.0-fold increased likelihood (95% CI 1.5–2.6) of manifesting anxiety symptoms and a 1.3-fold increased likelihood (95% CI 1.0–1.8) of being at risk for PTSD. Likewise, participants who had experienced the death of a close family member due to coronavirus infection exhibited a 1.7-fold increased likelihood (95% CI 1.2–2.4) of reporting depression symptoms. Moreover, they were 1.7 times more likely (95% CI 1.2–2.4) to experience elevated anxiety levels and 1.7 times more likely (95% CI 1.2–2.3) to be at risk for PTSD, even after adjusting for variables such as age, gender, and pre-existing mental health-related problems.

**Table 5:** Predictive factors of common mental health symptoms using regression analysis.

		Number (%)	Depression			Anxiety			PTSD		
			Odds Ratio (OR)^¥^	95% CI	*p-value*	Odds Ratio (OR)^¥^	95% CI	*p-value*	Odds Ratio (OR)^¥^	95% CI	*p-value*
**Student has any medical problems**	No	3486 (93.1)	1.0	Ref		1.0	Ref		1.0	Ref	
	Yes	259 (6.9)	1.2	0.8–1.7	0.19	**2.0**	**1.5–2.6**	**<0.01**	**1.3**	**1.0–1.8**	**0.002**
**Student is/got sick or is/was hospitalized because of the current coronavirus pandemic**	False	3419 (91.3)	1.0	Ref		1.0	Ref		1.0	Ref	
	True	203 (5.4)	1.3	0.8–1.8	0.16	1.1	0.7–1.5	0.54	1.1	0.8–1.6	0.25
	Not sure	123 (3.3)	1.3	0.7–2.4	0.24	1.3	0.8–2.2	0.21	0.8	0.5–1.2	0.35
**A close family member is/got sick or is/was hospitalized because of the current coronavirus infection**	False	3072 (82.0)	1.0	Ref		1.0	Ref		1.0	Ref	
	True	525 (14.0)	1.0	0.8–1.3	0.63	**1.3**	**1.0–1.6**	**<0.001**	1.0	0.8–1.3	0.38
	Not sure	148 (4.0)	0.6	0.3–1.1	0.11	0.6	0.4–1.0	0.10	1.0	0.6–1.4	0.99
**A close family member died because of the current coronavirus**	False	3367 (89.9)	1.0	Ref		1.0	Ref		1.0	Ref	
	True	216 (5.8)	**1.7**	**1.2–2.4**	**<0.01**	**1.7**	**1.2–2.3**	**<0.01**	**1.7**	**1.2–2.3**	**<0.01**
	Not sure	162 (4.3)	1.2	0.7–2.0	0.37	**1.6**	**1.0–2.4**	**<0.001**	1.2	0.8–1.8	0.23

## Discussion

This cross-sectional web-based study investigated the prevalence of mental health symptoms (depression, anxiety, and risk for PTSD) among 3,745 school students in the UAE post lockdown of the COVID-19 pandemic. This study also identified the potential factors that predicted school students’ depression, anxiety, and risk for PTSD.

### Prevalence of anxiety, depression, and risk for PTSD

This study has adequate evidence that students in the UAE are exhibiting symptoms of mental health disorders in the post COVID-19 pandemic period and this is consistent with that of recent studies across the globe. In a comprehensive review of evidence, the WHO concluded that the world has experienced a 25% increase in the prevalence of anxiety and depression in the first year of the COVID-19 crisis. More precisely, the report revealed that the pandemic has particularly affected the mental health of young people who, more seriously, were found disproportionally at risk of suicidal and self-harming behaviors [37]. Accordingly, the organization considered the COVID-19 pandemic a wake-up call to the world to set up mental health services [38]. Another study examined the mental health challenges among Bangladeshi healthcare professionals during COVID-19, urging comprehensive mental health support. This study may offer valuable context and a comparative viewpoint to better comprehend the mental health symptoms observed in school students in the UAE following the lockdown. It highlighted the significance of comprehensive mental health support and interventions, emphasizing the need for such measures not only during but also after the pandemic [39]. More support can be derived from a study that explored workplace stressors for nurses during the COVID-19 pandemic, emphasizing the need for comprehensive mental health interventions. Understanding these dynamics is critical for contextualizing and comparing the mental health experiences of school student’s post-lockdown in the UAE, emphasizing the need for planned interventions across various affected populations [40]. Several other studies indicated that adolescents experienced more adverse mental health effects during the pandemic in comparison to adults. These comparative findings underscored that adolescents were more prone to reporting symptoms of depression, anxiety, and post-traumatic stress disorder (PTSD) [41, 42].

### Gender and age differences

The results from this study highlight that female and older adolescents showed a significantly higher proportion of anxiety, depression, and risk for PTSD symptoms. These differences are supported by a number of recent studies that showed female high school students at an increased risk of psychological stress [43] and at higher levels of anxiety and depressive symptoms during the pandemic [44]. Interestingly, a number of researchers have argued that a possible explanation for female high school students’ higher levels of anxiety, depression, and stress symptoms during the COVID-19 pandemic could be due to physiologic hormonal and bodily changes or a lack of coping mechanisms [45, 46].

### Past medical history and family/social support

This study explored more associations between mental symptoms and existing medical conditions. Findings showed that students who had any medical problem were 2.0 (95% CI 1.5–2.6) times more likely to have anxiety, 1.3 (95% CI 1.0–1.8) times more likely to have PTSD. These results are supported by a recent post-lockdown study conducted in Germany which showed that children with complex chronic diseases were more likely to have mental health problems [47]. In another supportive study, researchers concluded that COVID-19-associated mental health risks were more likely to appear in children and adolescents with special needs [48]. Conventionally, social ties and family relationships are understood to have a significant impact on the maintenance of psychological well-being and reduce the risk for depression among adolescents [49–53]. The relevant findings from our study are supportive of this conviction; participants who had experienced the death of a close family member due to coronavirus infection were 1.7 (95% CI 1.2–2.4) times more likely to have depression, 1.7 (95% CI 1.2–2.3) times more likely to have anxiety, and 1.7 (95% CI 1.2–2.3) times more likely to have PTSD.

In a nutshell, symptoms of anxiety, depression, and risk for PTSD were found in the UAE school students. Mental health services are needed to help those children cope and recover as the Covid-19 pandemic is receding. Special attention must be regarded to disadvantaged children and proactive plans should be put in place for potentially forthcoming threats.

### Strengths and limitations

The study holds several strengths that underscore its significance and contribution to the field of children mental health research in the UAE. Interestingly, healthcare entities in the country have proactively prioritized mental health even before the pandemic, and the study takes advantage of this groundwork. The collaboration of a multi-disciplinary team consisting of government health regulators, disaster management experts, healthcare providers, and academia further enhances the robustness of the research. Notably, the involvement of parents and students from across the seven emirates strengthens the representativeness of the findings. The generation of robust datasets serves as a valuable resource for multiple disciplines, including physical and mental health, education, social studies, and economics, amplifying the study’s utility. An additional strength lies in the novelty of the study, as the literature review highlights its pioneering nature in the UAE context. However, the study is not without limitations. The wealth of data variables presents a challenge in comprehensive incorporation within a single study. This constraint, while inevitable, offers an avenue for subsequent research to extract relevant studies from the data bank, thus serving the broader community of mental health professionals and policymakers. Like many similar research efforts conducted during the ongoing pandemic, the challenge of relying on convenience and snowball sampling methods was inevitable [54]. Chosen for their practicality given the circumstances, it is important to acknowledge that this approach can have implications for the generalizability of our findings to the broader population.

## Conclusion

The global COVID-19 pandemic, characterized by widespread lockdowns and pervasive uncertainty, has inflicted significant mental health challenges upon children worldwide. Our study diligently examined the prevalent mental health symptoms experienced by UAE school students after the pandemic’s lockdown phase. It delved into anxiety, depression, and PTSD symptoms among students, uncovering crucial associations with demographic and health factors. Notably, late adolescents and females exhibited heightened vulnerability to various forms of mental distress. Additionally, students with pre-existing medical issues and those with family members affected by the virus faced significantly increased odds of mental symptoms. These results highlight the urgent necessity for tailored mental health support initiatives designed for students, with a specific focus on those at an elevated risk. Furthermore, this work underscores the critical importance of conducting subsequent studies to evaluate the pandemic’s enduring effects. Additionally, there is a vital need to incorporate a mental health support system into disaster preparedness plans, recognizing the significance of mental well-being in crisis management.

### Implications

The implications of our findings are substantial in shaping psychological support strategies for children. Specific attention must be given to vulnerable groups, such as late adolescents, female students, and those grappling with complex chronic illnesses or challenging family dynamics. Individuals who underwent pandemic-related traumas, like severe illness or bereavement, require targeted interventions. Continuous mental wellness evaluations, tailored coping mechanisms for evolving learning contexts, prompt identification of individual psychological needs, and accessible interventions for distressed students are essential. Educators need training to discern school students’ verbal and non-verbal cues indicating potential mental distress, fostering effective school-family communication. These insights underscore the importance of developing a comprehensive school mental health program that promotes the well-being of youth and enhances their resilience against future uncertainties.
