# Supplementary material for: Prevalence and factors associated with mental illness symptoms among school students post lockdown of the COVID-19 pandemic in the United Arab Emirates: A cross-sectional national study
Source: PLoS One. 2024 Feb 1;19(2):e0296479. doi: 10.1371/journal.pone.0296479 (PMC10833540; doi:10.1371/journal.pone.0296479)
Supplement: Attachment — Submitted filename: Response to Reviewers.docx [file pone.0296479.s001.docx]

Prevalence and Factors Associated with Mental Illness Symptoms among School Students Post Lockdown of the COVID-19 Pandemic in the United Arab Emirates: A Cross-Sectional National Study

**Dear Reviewers**

**On behalf of the research team, we would like to thank you for the very valuable feedback. Your suggestions are developmental and will definitely make our work immaculate. Please find our responses marked in olive green.**

Journal Requirements:

When submitting your revision, we need you to address these additional requirements.

1. Please ensure that your manuscript meets PLOS ONE's style requirements, including those for file naming. The PLOS ONE style templates can be found at

<https://journals.plos.org/plosone/s/file?id=wjVg/PLOSOne_formatting_sample_main_body.pdf> and

<https://journals.plos.org/plosone/s/file?id=ba62/PLOSOne_formatting_sample_title_authors_affiliations.pdf>

**The manuscript and other relevant documents were prepared per PLOS ONE's style requirements.**

2. Please provide additional details regarding participant consent. In the ethics statement in the Methods and online submission information, please ensure that you have specified what type you obtained (for instance, written or verbal, and if verbal, how it was documented and witnessed). If your study included minors, state whether you obtained consent from parents or guardians. If the need for consent was waived by the ethics committee, please include this information.

**Details on participant consent were added.**

- **They can be tracked in the manuscript (lines 146-147, 169-172).**
- **For the online submission information (online questionnaire) , details are found on the Participant Information Sheet, section “Participants’ role in the study”, sub-section “Part 1 (Questions 1-11)”**

3. We note that you have indicated that data from this study are available upon request. PLOS only allows data to be available upon request if there are legal or ethical restrictions on sharing data publicly. For more information on unacceptable data access restrictions, please see <http://journals.plos.org/plosone/s/data-availability#loc-unacceptable-data-access-restrictions>.

In your revised cover letter, please address the following prompts:

a) If there are ethical or legal restrictions on sharing a de-identified data set, please explain them in detail (e.g., data contain potentially sensitive information, data are owned by a third-party organization, etc.) and who has imposed them (e.g., an ethics committee). Please also provide contact information for a data access committee, ethics committee, or other institutional body to which data requests may be sent.

b) If there are no restrictions, please upload the minimal anonymized data set necessary to replicate your study findings as either Supporting Information files or to a stable, public repository and provide us with the relevant URLs, DOIs, or accession numbers. For a list of acceptable repositories, please see <http://journals.plos.org/plosone/s/data-availability#loc-recommended-repositories>.

**The below statement has been added to the revised cover letter:**

**“Anonymized data set necessary to replicate our study findings is made available on a public data repository (Figshare)**

[**https://doi.org/10.6084/m9.figshare.22231585**](https://doi.org/10.6084/m9.figshare.22231585)**)**

**The below statement was added to the manuscript (Lines 157-159). Reference (Ref # 17)**

**“Anonymized data set necessary to replicate the study findings is available to other researchers on a public repository [17].”**

We will update your Data Availability statement on your behalf to reflect the information you provide.

[Note: HTML markup is below. Please do not edit.]

Reviewers' comments:

Reviewer's Responses to Questions

**Comments to the Author**

1. Is the manuscript technically sound, and do the data support the conclusions?

The manuscript must describe a technically sound piece of scientific research with data that supports the conclusions. Experiments must have been conducted rigorously, with appropriate controls, replication, and sample sizes. The conclusions must be drawn appropriately based on the data presented.

Reviewer #1: Yes

Reviewer #2: Yes

2. Has the statistical analysis been performed appropriately and rigorously?

Reviewer #1: Yes

Reviewer #2: I Don't Know

**Data set is now accessible to reviewer for evaluation.**

3. Have the authors made all data underlying the findings in their manuscript fully available?

The [PLOS Data policy](http://www.plosone.org/static/policies.action#sharing) requires authors to make all data underlying the findings described in their manuscript fully available without restriction, with rare exception (please refer to the Data Availability Statement in the manuscript PDF file). The data should be provided as part of the manuscript or its supporting information, or deposited to a public repository. For example, in addition to summary statistics, the data points behind means, medians and variance measures should be available. If there are restrictions on publicly sharing data—e.g. participant privacy or use of data from a third party—those must be specified.

Reviewer #1: Yes

Reviewer #2: Yes

4. Is the manuscript presented in an intelligible fashion and written in standard English?

PLOS ONE does not copyedit accepted manuscripts, so the language in submitted articles must be clear, correct, and unambiguous. Any typographical or grammatical errors should be corrected at revision, so please note any specific errors here.

Reviewer #1: Yes

Reviewer #2: Yes

5. Review Comments to the Author

Please use the space provided to explain your answers to the questions above. You may also include additional comments for the author, including concerns about dual publication, research ethics, or publication ethics. (Please upload your review as an attachment if it exceeds 20,000 characters)

Reviewer #1: Dear PLOS ONE team of Editorials, thank you for the chance given to me to re-review “Deworming utilization among pregnant mothers with at least one Antenatal care follow up in Ethiopia, 2022: - A Multilevel analysis” with manuscript Number PONE-D-22-26022R1. I am thankful for addressing and correcting the [previous version of the manuscript. The following are my comments in this version;
1. In your response to my comment regarding regional deworming status you have responded as “Hence, we did not show it in the multivariable model t. Nevertheless, the highest prevalence is observed in Dire Dewa town (13.8%) and the lowest one in Somali region (4.4%)”. Therefore, try to also incorporate this in the manuscript as well since this is also one of the findings.
2. The mass campaign deworming Vs your response “ , those mothers with poor wealth index/ economic status will not routinely utilize health services due to various reasons like transportation issues, their poor knowledge regarding to such services etc. If the pregnant mother does seek health institutions regularly, she will not have a chance to get deworming and other complementary ANC services.” are contraindicating and have you analyzed it specifically? Otherwise, you were cross-reasoning like …poor poverty…NOT have chance to get deworming and OTHER?
3. Try to ensure all the sections for their main and key contents.
4. Reference your classifications well e.g., educational level (High and Low), Income…etc.
5. Avoid repetition and maintain consistency particularly on scientific rigor.
6. Minor language, full stop missing, capital letters and abbreviations, and statistical corrections are needed e.g. Moslem to mean Muslim and Addis Abeba to mean Addis Ababa, catholic instead of Catholic, statistics not beginning from higher to lower.
7. Meticulous proof read is needed before the actual publication.

**This review is irrelevant to our study.**

Reviewer #2: Thank you for inviting me to review this article. This is a study on the prevalence of mental health symptoms and its associated factors among a large sample of children and adolescents in the UAE. I enjoyed reading the article and have several suggestions to improve it.

There were contradictory statements regarding the data availaibility. ‘Yes - all data are fully available without restriction’, / Data are available from the Emirates Health Services Institutional Data Access / Ethics Committee (contact via [MHD@ehs.gov.ae](mailto:MHD@ehs.gov.ae)) for researchers who meet the criteria for access to confidential data.

**Data availability was corrected in the cover letter and the manuscript (Lines 157-159)**

INTRODUCTION

Paragraph 2

There has been lack of reference to the current state of evidence regarding the impact of the pandemic on the mental health of children and young people. What has systematic reviews/meta-analyses within this topic has found? I suggest for authors to conduct a simple literature search to find relevant reviews on this topic.

Search string that can be used e.g ‘systematic review’ AND ‘mental health’ AND COVID-19 AND (‘young people’ OR children OR adolescent) and you can find several related reviews

Some recently published examples that might be relevant for consideration:

Mental health changes before vs during the pandemic: <https://doi.org/10.1007/s00787-022-02060-0>

Impact on clinical groups: <https://doi.org/10.1177/20503121221086712>

Prevalence and correlates of mental health: <https://doi.org/10.1080/23311908.2022.2111849>

**Data from a systematic review were added (lines 58-62). Reference (Ref # 4)**

Paragraph 3-4

There appears to be a number of related studies in UAE already so I suggest a brief sentence/sentences that highlight how this current study build on these already published works.

**Two studies from the UAE were added (lines 92-96). References (Ref # 10 and 11)**

METHODS

Can you clarify the total population of the students and sampling strategy used? While it is clear n=xx were eventually recruited, how many participants were invited? It seems that all private and public schools in UAE were invited therefore it might be possible to obtain the numbers and estimate total population and how the sample was obtained.

**The invite was sent out to all students in private and public schools aged 8-18. The total population has been estimated at about 721048 per the UAE Open Data Portal https://data.government.ae/dataset?tags=ODT&tags=education&res_format=XLSX&organization=ministry-of-education. Total population sampling was used to obtain the sample.**

**Please note that the above explanation was added in the manuscript (lines 127-129). Reference (Ref # 12)**

I notice the authors define the age groups into; Pre-adolescence (<10 years - ?what’s the minimum age) **minimum age is 8**, Early adolescence (10-13 years), Middle adolescence (14-17 years) and Late adolescence (18 year). There has been variations of age groups definition and terminologies for children, adolescent and young people etc. Can you share any reference or justification for the age group division that you used? Are these age groups relate to the grades/schooling system in the UAE?

**Age groups were identified based on two resources:**

1. **the UAE stages of school education**

**Stages and streams of school education - the official portal of the UAE Government. Retrieved March 6, 2023, from https://u.ae/en/information-and-services/education/school-education-k-12/joining-k-12-education/stages-and-streams-of-school-education**

2. **Published Article:**

**Salmela-Aro, K. (2011). Stages of adolescence. *Encyclopedia of Adolescence*, 360–368. https://doi.org/10.1016/b978-0-12-373951-3.00043-0**

**A note on the age group was added to the manuscript (Lines 198-199). References (Ref # 18 and 19)**

RESULTS

It was clarified that for table 1 ‘Percentages may not add up to 100 because of missing data’. I suggest the descriptive amount and percentage of missing data to be included in the table.

**Data on table 1 (total and missing entries) were added.**

STRENGTHS AND LIMITATIONS

There was mentioned about a pilot test being done which i did not find earlier. I suggest to describe the details of the pilot test in Methods. Worth noting that the study is observational therefore unable to make causation of the pandemic > mental health, and cross-sectional so unable to ascertain whether it got worse than before pandemic. It was also a bit unclear whether the large sample size can be generalized to the nationwide population level, given unclear if it uses probability vs nonprobability sampling method.

**Pilot test was added to the method section (Lines 152-153).**

6. PLOS authors have the option to publish the peer review history of their article ([what does this mean?](https://journals.plos.org/plosone/s/editorial-and-peer-review-process#loc-peer-review-history)). If published, this will include your full peer review and any attached files.


If you choose “no”, your identity will remain anonymous but your review may still be made public.


**Do you want your identity to be public for this peer review?** For information about this choice, including consent withdrawal, please see our [Privacy Policy](https://www.plos.org/privacy-policy).

Reviewer #1: No

Reviewer #2: No
