# Supplementary material for: Prevalence and factors associated with mental illness symptoms among school students post lockdown of the COVID-19 pandemic in the United Arab Emirates: A cross-sectional national study
Source: PLoS One. 2024 Feb 1;19(2):e0296479. doi: 10.1371/journal.pone.0296479 (PMC10833540; doi:10.1371/journal.pone.0296479)
Supplement: Attachment — Submitted filename: Response to Reviewers (29-08-2023).docx [file pone.0296479.s002.docx]

Prevalence and Factors Associated with Mental Illness Symptoms among School Students Post Lockdown of the COVID-19 Pandemic in the United Arab Emirates: A Cross-Sectional National Study

**Dear Reviewers**

**We wish to express our gratitude to you for dedicating your time and expertise to review our work. Your meticulous evaluation and perceptive feedback have played an integral role in refining and strengthening our study. Here is the team’s response to your comments and suggestions marked in bold brown font.**

Journal Requirements:

Please review your reference list to ensure that it is complete and correct. If you have cited papers that have been retracted, please include the rationale for doing so in the manuscript text, or remove these references and replace them with relevant current references. Any changes to the reference list should be mentioned in the rebuttal letter that accompanies your revised manuscript. If you need to cite a retracted article, indicate the article’s retracted status in the References list and also include a citation and full reference for the retraction notice.

**The reference list was updated.**

Additional Editor Comments (if provided):
Dear Author, reviewers still have some concerns as a mix of major and minor comments. Could you please try to address and return the manuscript with your revise version? Thank you!

**All reviewers’ concerns were taken into consideration and edits were introduced accordingly.**

[Note: HTML markup is below. Please do not edit.]

Reviewers' comments:

Reviewer's Responses to Questions

**Comments to the Author**

1. If the authors have adequately addressed your comments raised in a previous round of review and you feel that this manuscript is now acceptable for publication, you may indicate that here to bypass the “Comments to the Author” section, enter your conflict of interest statement in the “Confidential to Editor” section, and submit your "Accept" recommendation.

Reviewer #1: All comments have been addressed

Reviewer #3: (No Response)

Reviewer #4: (No Response)

Reviewer #5: (No Response)

2. Is the manuscript technically sound, and do the data support the conclusions?

The manuscript must describe a technically sound piece of scientific research with data that supports the conclusions. Experiments must have been conducted rigorously, with appropriate controls, replication, and sample sizes. The conclusions must be drawn appropriately based on the data presented.

Reviewer #1: No

Reviewer #3: Yes

Reviewer #4: Partly

Reviewer #5: Partly

3. Has the statistical analysis been performed appropriately and rigorously?

Reviewer #1: No

Reviewer #3: Yes

Reviewer #4: Yes

Reviewer #5: No

4. Have the authors made all data underlying the findings in their manuscript fully available?

The [PLOS Data policy](http://www.plosone.org/static/policies.action#sharing) requires authors to make all data underlying the findings described in their manuscript fully available without restriction, with rare exception (please refer to the Data Availability Statement in the manuscript PDF file). The data should be provided as part of the manuscript or its supporting information, or deposited to a public repository. For example, in addition to summary statistics, the data points behind means, medians and variance measures should be available. If there are restrictions on publicly sharing data—e.g. participant privacy or use of data from a third party—those must be specified.

Reviewer #1: Yes

Reviewer #3: Yes

Reviewer #4: Yes

Reviewer #5: Yes

5. Is the manuscript presented in an intelligible fashion and written in standard English?

PLOS ONE does not copyedit accepted manuscripts, so the language in submitted articles must be clear, correct, and unambiguous. Any typographical or grammatical errors should be corrected at revision, so please note any specific errors here.

Reviewer #1: Yes

Reviewer #3: No

Reviewer #4: Yes

Reviewer #5: Yes

6. Review Comments to the Author

Please use the space provided to explain your answers to the questions above. You may also include additional comments for the author, including concerns about dual publication, research ethics, or publication ethics. (Please upload your review as an attachment if it exceeds 20,000 characters)

Reviewer #1: Review Report
Title: Prevalence and Factors Associated with Mental Illness Symptoms among School Students Post Lockdown of the COVID-19 Pandemic in the United Arab Emirates: A Cross-Sectional National Study.
Manuscript Number: PONE-D-22-19659R1
Review Comments
1. General Comments
♣ Is that the prevalence of mental illness or prevalence mental illness symptoms?

**The study examined the prevalence of mental illness symptoms.**

Why only among school students? What was unique among them since the pandemic have some sort of mental effect on the entire population? Why after the lockdown?

**Although the pandemic is sought to have impacted the entire population, this study focused on particularly vulnerable groups (children and adolescents) who are at crucial stages of development, both cognitively and emotionally. The pandemic-related stressors, such as isolation, online learning challenges, and fear of the virus, could have lasting effects on their mental well-being. As for why it is after lockdown, the mental health effects of the pandemic might not manifest immediately; they could have long-term implications on children's psychological well-being. By assessing mental health post-lockdown, we can capture both immediate reactions and potential delayed responses, ensuring a comprehensive understanding of the situation.**

♣ Since it is self-report [self-diagnosis] what can the clinicians or the health system benefited?

**Clinicians can potentially identify early signs of mental health concerns and implement timely interventions. This preventive approach aligns with the broader goal of promoting mental well-being from a young age and reducing the likelihood of more severe mental health issues later in life.**

♣ Is that common mental health illnesses or special type of mental illnesses that had occurred or anticipated to occur secondary to COVID 19?

**Anxiety, depression, and post-traumatic stress disorder (PTSD) are all common mental disorders; however, the abrupt transition from lockdown to a more open society can create stress as children and adolescents adapt to changes in routines, social interactions, and expectations. The uncertainty of this transition can contribute to heightened mental challenges especially to those who directly experienced illness or loss due to COVID-19.**

♣ All the sections of the manuscript are inadequate for its scientific contents.

**The scientific content was revisited and significant additions were introduced.**

2. Specific Comments
¬ Establish the niche more and try to cite the UN and national recommendations in the background section.

**International perspectives and recommendations were added, please check lines 42, 44, 47, 58, 60, 69, and 73**

¬ If web based, its likely that less costly. Hence, why don’t you include other segment of the population?

**Although authors had accessibility to the entire population, this study sought to focus on school children due to the vulnerability of this age group.**

¬ How do you ensure that huma being had responded to your questions?

**Respondents were expected to be human beings because the questionnaire link was sent out via the participating school administrations and other media means to the public.**

¬ What are the steps taken to ensure data quality measures, when and how?

**Ensuring data quality remained paramount in our study. Collaborative data handling involving multiple authors added rigor by enabling cross-validation and error minimization. Complementing this, a dependable data analysis software, IBM SPSS Statistics 26, was employed. This software not only facilitated streamlined analysis but also enhanced the consistency and reliability of our results. Additionally, our data collection was conducted using a government-powered platform (mSurvey), which provided standardized templates and guaranteed data protection and privacy, reinforcing the ethical foundation of our study.**

¬ Have you fitted the model and its indicators were not described.

**In this study, the authors had the following objectives:**

1. **To determine the prevalence rates of depression, anxiety, and risk for PTSD among school students in the UAE post-COVID-19 lockdown through descriptive statistics.**
2. **To investigate the associations between socio-demographic variables (age group, gender) and other pandemic-related factors (e.g., family member experiences) with the presence of mental health symptoms**
3. **To build predictive models that identify significant predictors (e.g., age, gender, pandemic-related experiences) of depression, anxiety, and risk for PTSD among students.**

**Here is how we structured the statistical model for this data analysis:**

**Outcome Variables:**

- **Depression (binary outcome: 0 = No, 1 = Yes)**
- **Anxiety (binary outcome: 0 = No, 1 = Yes)**
- **Risk for PTSD (binary outcome: 0 = No, 1 = Yes)**

**Predictor Variables:**

- **Age Group (categorical: pre-adolescents, early adolescence, middle adolescence, late adolescence)**
- **Gender (binary: 0 = Female, 1 = Male)**
- **Medical Problems (binary: 0 = No, 1 = Yes)**
- **Close Family Member Sick/Hospitalized due to Coronavirus (binary: 0 = No, 1 = Yes)**
- **Close Family Member Died due to Coronavirus (binary: 0 = No, 1 = Yes)**
- **School Attendance (binary: 0 = Online, 1 = In-person)**

**Statistical Analysis Steps:**

- **Descriptive Statistics: We calculated frequencies and percentages for socio-demographic characteristics, pandemic-related factors, and mental health symptoms.**
- **Inferential analysis:**
- **We used Chi-squared tests (χ²) to evaluate associations between categorical variables**
- **P-values were computed to determine the significance of associations.**
- **We conducted separate logistic regression models to explore the influence of various categorical variables on the presence of symptoms (Depression, Anxiety, PTSD) generating Odds Ratios (OR), 95% Confidence Intervals (Cis), and P-values to quantify the impact of independent variables on the presence of symptoms.**

Top of Form

¬ How did you manage those with ‘mental health illnesses?

**As the study did not involve the reference to medical diagnosis, all children and adolescents who responded were included.**

Is there respondents aged less than 18 years old? Hence, how did you approach? Did they signed electronic signature?

**The study population included children and adolescents aged 8-18 years (Please see the method section). Invites for participation were sent out to the public via multiple corporate channels including SMS, press release, and social media platforms. In addition, school-parent communication channels served as a fundamental resource for reaching out to the study participants. As is conventional in anonymous surveys, consent was in place before starting the survey. Parents confirmed their consent in the electronic questionnaire by answering two written mandatory questions placed just at the end of the Participant Information Page. Students themselves were also requested to confirm their consent by responding to one mandatory written question just before they answered the electronic survey questions (please refer to the ethical consideration section lines 149-162).**

¬ Is that descriptive or analytic study?

**The study incorporates both descriptive elements to paint a detailed picture of the participants and analytical elements to explore relationships and draw meaningful conclusions from the data.**

¬ The result should be in line with the study objective?

**The study objectives were re-phrased to show the alignment (Please see lines 93-98.)**

¬ The results should entail what it intends to entail in scientific paper. Similarly, address for the discussion and conclusion section. Try also to draw implications.

**Modifications were introduced (Please check findings-line 180 and implications-line 305 sections)**

¬ Include the complete key words and avoid abbreviations without description in the abstract.

**Done**

¬ Include International agency references E.g., WHO, IHME/GBD, UAE national health policy and strategy and COVID-19 protocol. Revisit your references.

**International perspectives and recommendations were added, please check lines 42, 44, 47, 58, 60, 69, and 73. Also please check references section.**

¬ Where are the acknowledgement, conflict of interest, funding and consent to publish in the manuscript?

**Please see the below excerpt from the “Participants Information Sheet” in the study questionnaire.**

Regards,

Reviewer #3: The following study, entitled 'Prevalence and Factors Associated with Mental Illness Symptoms among School Students Post Lockdown of the COVID-19 Pandemic in the United Arab Emirates: A Cross-Sectional National Study,' investigated the prevalence of mental illness symptoms (depression, anxiety, and PTSD) among school students in the UAE after the COVID-19 pandemic lockdown. The study is relevant, but there are several points that should be considered.


1- The author needs to remove the subheading of the objective as it is part of the introduction.

**Done** (**please see line 93)**

2- The following sentence, in lines 101-103, is not clear: 'Parents of the surveyed students participated in the study by consenting on behalf of their under-age children and answering a series of questions within the same instrument.' Could you please clarify what the author means by 'under-age children'?

**In this context, “under-age” means minors or children below 18 years old (per UAE Federal Law No. 3 of 2016 (Wadeema’s Law). Under-age was changed to children.**

3- In method A, the paragraph (lines 158 to 161) is not clear. If the author used logistic regression to associate the status of having symptoms (nominal data; yes or no) with other student factors, it is unclear why there is a mention of the Pearson's coefficient test. Additionally, it would be better to rephrase 'Multiple linear regression' as follows: 'Multiple linear regression analyses were performed to examine the association between students' mental illness symptom scores and selected demographic and COVID-19 factors.'

**Correction done (please see the modified data analysis section)**

4- It is not clear from the findings who the participants are: whether they are the students themselves or the parents of the students. Please provide further clarification on this matter.

**Parents answered parts 1 and 2 of the questionnaire, while students answered part 3. (Please see the below excerpt from the study questionnaire).**

5- If students responded to the questionnaire, please mention them in the table under the column 'Person Responding to this Questionnaire'.

**Done (please see lines 181, 191, and 199)**

6- The study's conclusion should not be more than one paragraph. Could you please summarize it?

**Done (please revisit the conclusion section)**

Reviewer #4: I had the opportunity to thoroughly review the revised version of the manuscript, and I would like to commend the authors for their diligent efforts. Overall, the authors have made significant improvements to the paper. In the following section, I provide constructive feedback and suggestions to further enhance the quality of the manuscript.
Abstract:
1. Please indicate what PTSD is.

**Done (Please see line 24)**

2. Based on Table 3, please check this sentence: "For gender differences, symptoms of the three conditions were higher in female students by 6.9%."

**Correction done, please check line 29.**

3. According to Table 5, the following sentence needs to be re-written correctly by either removing the values or adding three values for each condition: "Further analysis revealed that having medical problems (OR = 2.0, p < 0.001) and witnessing the death of a close family member due to COVID-19 (OR = 1.7, p < 0.001) were positive predictors associated with PTSD, depression, and anxiety."

Additionally, OR should be used instead of β.

**Done (Please see lines 31-33)**

Introduction:
1. Please add references to the sentences ending in lines 43, 53, and 55.

**Done (Please see lines 39-56)**

2. Lines 135-137 and 152 have a different font and size than the rest of the paper.

**Done**

Methods:
1. I am a bit confused regarding data ownership. Do the authors of this paper own the data, or is this study a secondary data analysis of a national survey?

**This study uses primary data collected by the study authors.**

2. What is your response rate?

# **In comparison to other similar studies done in the UAE, our response rate is considered relatively high. In the below mentioned UAE national studies, convenient samples of 1002, 4,426, 798 participants were included respectively. Similarly, our survey aimed to capture insights from the entire student population, which consists of a diverse and expansive group. Given the scope of our study and the logistical challenges of reaching every student across the country especially at that critical period of schools reopening, convenience and snowball sampling techniques were used.**

**Abdelrahman R, Ismail ME. The psychological distress and covid-19 pandemic during lockdown: A cross-sectional study from United Arab Emirates (UAE). Heliyon. 2022;8(5). doi:10.1016/j.heliyon.2022.e09422**

**Cheikh Ismail L, Mohamad MN, Bataineh MF, Ajab A, Al-Marzouqi AM, Jarrar AH, et al. Impact of the coronavirus pandemic (COVID-19) lockdown on mental health and well-being in the United Arab Emirates. Frontiers in Psychiatry. 2021;12. doi:10.3389/fpsyt.2021.633230**

**Vajpeyi Misra A, Mamdouh HM, Dani A, Mitchell V, Hussain HY, Ibrahim GM, et al. Impact of covid-19 pandemic on the mental health of university students in the United Arab Emirates: A cross-sectional study. BMC Psychology. 2022;10(1). doi:10.1186/s40359-022-00986-3**

3. Based on the numbers mentioned in this paper, you have a very low response rate. Therefore, it is important to ask why the authors decided to close/end the survey. Why was the survey extended only for six months despite the low response rate?

**While a longer survey duration could have yielded a higher response rate, the decision to open the survey for six months post COVID-19 lockdown was a careful balance between the desire for timeliness, contextual relevance, and the need to minimize potential biases introduced by a more extended period. In other words, a shorter data collection period aimed to minimize potential recall bias. As the pandemic's impact evolved, respondents' recollections of their mental health experiences could have been influenced by various external factors, thereby introducing recall bias. By keeping the survey period close to the lockdown, we aimed to capture more immediate, unaltered reflections of participants' experiences.**

4. What strategy was implemented to increase the response rate? Are there any other studies in the UAE with a similar response rate?

**We employed several strategies to encourage participation. These included clear and engaging survey communications, reminders, and informative content highlighting the study's significance. As for similar studies in the UAE, here are again a list of 3 studies that used convenience and snowball sampling techniques of 1002, 4,426, 798 participants.**

**Abdelrahman R, Ismail ME. The psychological distress and covid-19 pandemic during lockdown: A cross-sectional study from United Arab Emirates (UAE). Heliyon. 2022;8(5). doi:10.1016/j.heliyon.2022.e09422**

**Cheikh Ismail L, Mohamad MN, Bataineh MF, Ajab A, Al-Marzouqi AM, Jarrar AH, et al. Impact of the coronavirus pandemic (COVID-19) lockdown on mental health and well-being in the United Arab Emirates. Frontiers in Psychiatry. 2021;12. doi:10.3389/fpsyt.2021.633230**

**Vajpeyi Misra A, Mamdouh HM, Dani A, Mitchell V, Hussain HY, Ibrahim GM, et al. Impact of covid-19 pandemic on the mental health of university students in the United Arab Emirates: A cross-sectional study. BMC Psychology. 2022;10(1). doi:10.1186/s40359-022-00986-3**

5. All these questions lead me to ask, how did you calculate your sample size?

**A convenience and snowball sampling techniques were adopted. A data collection period was specified to be the closest to the post-lockdown period, and all participants who responded fully to the questionnaire were included.**

6. Please provide more information on your pilot study. How did you assess the reliability and validity of both surveys as mentioned in the methods section?

**The questionnaire contained questions that collected biodata and 3 standardized scales (MFQ-Child self-report, SCARED-Child Version, and CRIES 8-Child Revised Impact of Events Scale). To ensure that the instrument was user-friendly and no technical difficulties were potentially existing, we ran 2 rounds of pilot testing. A group of bilingual students and parents were asked to respond anonymously to the online questionnaire and their feedback was collated to introduce modifications on the participant information sheet and the biodata questions.**

7. It seems that your survey was administered in two languages (English and Arabic). How was the survey translated, and did you perform back translation?

**The questionnaire used standardized scales that are already available in both languages (Arabic and English) and tested for reliability and validity.**

**Children and War Foundation. Measures-Children’s Impact of Event Scale-CRIES-8 [Internet]. 2022 [cited 2023 Sept 4]. Available from: https://www.childrenandwar.org/wp-content/uploads/2019/06/Childrens-Impact-of-Event-Scale-CRIES-8-ARABIC.pdf**

**Tavitian L, Atwi M, Bawab S, Hariz N, Zeinoun P, Khani M, et al. The Arabic mood and feelings questionnaire: Psychometrics and validity in a clinical sample. Child Psychiatry &amp; Human Development. 2013;45(3):361–8. doi:10.1007/s10578-013-0406-6**

**Hariz N, Bawab S, Atwi M, Tavitian L, Zeinoun P, Khani M, et al. Reliability and validity of the Arabic screen for child anxiety related emotional disorders (scared) in a clinical sample. Psychiatry Research. 2013;209(2):222–8. doi:10.1016/j.psychres.2012.12.002**

Results:
1. Please add a zero in front of the decimal.

**Done**

2. Report the exact p-value as it appeared in the SPSS output, even without rounding.

**Done**

Discussion:
1. I don't agree with this statement, given the low response rate: "This study has adequate evidence that students in the UAE are exhibiting symptoms of mental health disorders in the post COVID-19 pandemic period, and this is consistent with recent studies across the globe."

**While response rate is a consideration, our study's approach, coupled with global trends, substantiates the assertion of mental health symptoms among UAE students post COVID-19. However, we appreciate your concern and in this revised manuscript, we will transparently discuss limitations tied to the response rate and their potential implications on generalizability (line 290). Here is a supportive global study:**

**Xiong, J., Lipsitz, O., Nasri, F., Lui, L. M. W., Gill, H., Phan, L., ... & McIntyre, R. S. (2020). Impact of COVID-19 pandemic on mental health in the general population: A systematic review. Journal of affective disorders, 277, 55-64.**

2. The authors can't generalize their findings with this very low response rate unless they applied survey weights in their statistical analysis.

**While survey weights were not applied in our analysis, we believe that the impact on our results is limited. Our study's comprehensive population-based approach aimed to capture a diverse cross-section of students in the UAE, encompassing a wide range of socio-demographic characteristics. As such, we are confident that our findings provide meaningful insights into the mental health landscape among students during the post-COVID-19 pandemic period. We value your input and will ensure to transparently discuss this aspect in our manuscript to provide a well-rounded understanding of our research (Please see lines 287-290).**

3. Since it is a national survey, I am surprised that the statistical team didn't create survey weights.

**While applying survey weights would have been no doubt helpful, none of the similar studies conducted in the UAE mentioned that survey weights were applied despite them using a similar range of participants. However, this perspective is addressed in the limitations section.**

4. Please include other limitations of your study, such as being a cross-sectional study, a hypothesis generating study and the possibility of socially desirable answers and recall bias.

**Other limitations are added (Please see lines 284-290)**

Conclusion:
The conclusion is too long. I suggest having a separate paragraph for the conclusion, and you might add a title for policy implications.

**Conclusion is rewritten.**

Reviewer #5: This study investigated the mental health symptoms and psychological distress experienced by school students in the United Arab Emirates during post lockdown of the COVID-19 pandemic. The idea was good; however, as it has been passed more than three years since the pandemic, this research can minimally be contributive to future decision-making in the respective country or region. Because the presented findings in the study have been reported in several studies before, and these kinds of findings have been well established.

However, after carefully reading the whole manuscript, I feel that this manuscript needs a major revision to achieve the merit of publication in a high-quality journal.

1. There are a few typos in the manuscript. Please go through the manuscript and correct them. E.g., in the abstract: PTDS.

**Edits done**

2. Write the full form of PTSD in the abstract on its first appearance.

**Done**

3. Rephrase the conclusion section of the abstract. It seems to me that the authors are summarizing another article. Write it in an active voice and in such a way that you performed this research.

**Done**

4. At this time, there are plenty of work have been conducted on this topic, and this type of research has little implication considering the stage of covid-19. Discuss more about the justification of this study at this time and how this study is different from the previous studies conducted in the UAE on the same/similar topic. What are the limitations of the previous studies?

**Kindly note that the study review process was delayed by PLOS One for a full year. Despite the inconvenience this slow process has caused to authors, we are still confident that the study holds its unique contributions, particularly given the absence of similar studies conducted in the UAE. Moreover, in this second review, we have incorporated additional relevant references to further enrich the study's context.**

5. The authors are suggested to elaborate on the data collection section. The authors reported that they invited all the students in the country (nearly one million students). How do they find the number to send SMS; responding to the press release, how the participants participated in the research; what social media platforms were used and how?

**For participant recruitment, we adopted a comprehensive strategy. In the initial phase, we engaged potential participants through school and parental communication avenues. Furthermore, we harnessed the electronic communication platforms affiliated with the authors' corporate connections. This entailed disseminating the invitation and study link to the public across official websites, press releases, and various social media platforms such as Facebook, Instagram, and Twitter. For SMS communication, we utilized the established networks of corporate facilities, including hospitals and clinics, to circulate the invitation to the employees’ families who met the inclusion criteria. This creative dissemination method facilitated broader access to our study's invitation.**

6. How the authors tested the validity and reliability of the questionnaire in the studied sample? Had these instruments been validated in the previous studies?

**All used scales are standardized (Mood and Feelings Questionnaire (MFQ)-Child self-report, Screen for Child Anxiety Related Disorders (SCARED)-Child Version, and Child Revised Impact of Events Scale (CRIES).**

7. Please elaborate on the scale ranges of the instruments and their cut-off values.

**Done. Please check lines 198-204**

8. I couldn’t understand what does mean this part, “a portal for remote counseling services was communicated to all participants”?

**A professional mental health support hotline was made accessible for participants in case they needed relevant support (Please see lines 161-162). Please check the below excerpt from the study questionnaire.**

9. Please replace the Table 2 title with a better one, “Table 2: Epidemic related information for the sample”. Epidemic could be replaced by covid-19.

**Done**

10. Line 164, what does mean by ‘expat’ here?

**In this context, expat means non-Emirati nationals.**

11. Write the full form of years.

**Modifications done**

12. I am not clear about the analysis of the data. What does mean by this line, “multivariate analysis was conducted to examine the associations between mental illness symptoms and related factors by using the Pearson’s product-moment correlation coefficient test? I don’t see any such analysis in the manuscript. Were the outcome variables binary variables? If so, how were they categorized? No description in the method section. Reporting of analyses performed and the presentation of findings are quite contradictory. The authors reported multiple linear regression; however, the presentation in Table 5 and their interpretation in the result section suggest they performed a logistic regression. Again, their interpretations in the abstract are different. Reported beta coefficient. Quite unclear to me.

**Data analysis section is re-written for clarification.**

13. Authors are suggested to report the outcome variables among the total/overall sample also in table 3, along with the gender and age differences.

**Done (Please check lines 198-204)**

14. The discussion section is too brief. Elaborate the discussion section and make comparisons with similar studies. Suggested articles that could be helpful.

**Thank you for providing these references. We have carefully reviewed the discussion sections in the references you suggested and made relevant edits to the corresponding section in our study accordingly.**

Chowdhury SR, Sunna TC, Das DC, Kabir H, Hossain A, Mahmud S, Ahmed S. Mental health symptoms among the nurses of Bangladesh during the COVID-19 pandemic. Middle East Curr Psychiatry. 2021;28(1):23. doi: 10.1186/s43045-021-00103-x.

Chowdhury SR, Kabir H, Mazumder S, Akter N, Chowdhury MR, Hossain A. Workplace violence, bullying, burnout, job satisfaction and their correlation with depression among Bangladeshi nurses: A cross-sectional survey during the COVID-19 pandemic. PLoS One. 2022 Sep 22;17(9):e0274965. doi: 10.1371/journal.pone.0274965.

Kabir H, Nasrullah SM, Hasan MK, Ahmed S, Hawlader MDH, Mitra DK. Perceived e-learning stress as an independent predictor of e-learning readiness: Results from a nationwide survey in Bangladesh. PLoS One. 2021 Oct 28;16(10):e0259281. doi: 10.1371/journal.pone.0259281.

Kabir H, Hasan MK, Mitra DK. E-learning readiness and perceived stress among the university students of Bangladesh during COVID-19: a countrywide cross-sectional study. Ann Med. 2021 Dec;53(1):2305-2314. doi: 10.1080/07853890.2021.2009908.

7. PLOS authors have the option to publish the peer review history of their article ([what does this mean?](https://journals.plos.org/plosone/s/editorial-and-peer-review-process#loc-peer-review-history)). If published, this will include your full peer review and any attached files.


If you choose “no”, your identity will remain anonymous but your review may still be made public.


**Do you want your identity to be public for this peer review?** For information about this choice, including consent withdrawal, please see our [Privacy Policy](https://www.plos.org/privacy-policy).

Reviewer #1: No

Reviewer #3: **Yes:** Fatemeh Sharif-Askari

Reviewer #4: **Yes:** Mona Abdelrehim

Reviewer #5: No
