# Supplementary material for: Prevalence and factors associated with mental illness symptoms among school students post lockdown of the COVID-19 pandemic in the United Arab Emirates: A cross-sectional national study
Source: PLoS One. 2024 Feb 1;19(2):e0296479. doi: 10.1371/journal.pone.0296479 (PMC10833540; doi:10.1371/journal.pone.0296479)
Supplement: Attachment — Submitted filename: Response to reviewers(31-10-2023).docx [file pone.0296479.s003.docx]

Dear Reviewers,

Thank you again for your valuable feedback. Please find below the modifications we introduced based on your comments.

Regards,

**Reviewer #1: The manuscript still fails to;**

**1.have stronger background for its update and rationale**

The introduction section was revisited and additional details were introduced.

**2. Assemble the fragments of ideas**

We have diligently worked on assembling fragmented ideas to create a cohesive and logical flow into the introduction of the manuscript. We ensured a smooth transition between sections and ideas, improving the overall readability and comprehension of the content.

**3.Clarity of the sub sections**

We acknowledge the need for enhancing clarity in sub-sections. We have revised and organized sub-sections in the method section to provide a clear and coherent structure, ensuring that each subsection has a distinct focus and effectively contributes to the narrative of the manuscript.

**4. Grammar and language flows**

We carefully reviewed and edited the manuscript to refine the grammar and language flow. Attention was given to sentence structure, word choice, and coherence to ensure a clear and engaging presentation of the research.

**5. Singular Vs plural e.g. method**

We have meticulously reviewed and ensured the consistent usage of singular or plural forms, as appropriate, throughout the manuscript, paying close attention to maintaining clarity and precision in conveying our intended meaning.

**6. Update the references**

In the revised version of the manuscript, we have meticulously cross-verified all references to ensure they are current and relevant to the subject matter. The inclusion of older references in this study is primarily tied to the use of well-established evidence-based questionnaires, which have stood the test of time in terms of reliability, validity, and acceptance within the academic and professional communities. These questionnaires, designed to measure specific constructs or variables, have become gold standards in their respective domains and have been extensively validated over the years.

**7. The tables and figures should be self-explanatory**

We fully agree with the importance of making our tables and figures self-explanatory to enhance the clarity and comprehensibility of the research presented. In our revised manuscript, we have taken the following measures to address this concern:

**Improved Captions and Detailed Legends:**

We have ensured that each table is accompanied by a clear and informative caption that succinctly explains the content and purpose. The captions is crafted to provide sufficient context for readers to interpret the data and findings without necessarily referring to the main text.

8**. Flawed use of cut off points e.g. p value less than 0.05**

SPSS, like other statistical software, typically reports p-values to three decimal places. However, it is important to note that this level of precision does not imply a higher level of significance. P-values below 0.05 are often reported as "< 0.001" or "< .001" for practical reasons related to formatting and readability. The Exact p- values in table 3 are added.

**9.Ensure the completeness of the sub sections on details**
We have carefully reviewed and enhanced the completeness of all subsections to ensure that they contain adequate and comprehensive information, providing readers with a thorough understanding of the content.

Regards,

**Reviewer #4: I would like to thank the authors for addressing the reviewers' comments.**

The following are my comments:

1. Although the authors mentioned using a convenient snowball sampling technique, they still need to determine the minimum number of respondents required to conduct multiple regression analyses.

For snowball sampling, the sample size is often not pre-determined with a specific formula. Instead, the sample size grows organically as new participants are referred by the initial participants.

Please refer to the following paper:

|  | **Age of student (years)** | | | | | **Gender of student** | | |
| --- | --- | --- | --- | --- | --- | --- | --- | --- |
|  | Pre-adolescence (<10 years)  [n=1285] | Early adolescence (10-13 years)  [n=1403] | Middle adolescence (14-17 years)  [n=888] | Late adolescence (18 years)  [n=67] | *p*-value | Male  [n=1895] | Female  [n=1800] | *p*-value |
| **Symptoms of one condition** |  |  |  |  |  |  |  |  |
| Depression only | 218 (17.0) | 246 (17.5) | 153 (17.2) | 18 (26.9) | 0.22 | 301 (15.9) | 333 (18.5) | <0.05 |
| Anxiety only | 288 (22.4) | 328 (23.4) | 219 (24.7) | 16 (23.9) | 0.68 | 377 (19.9) | 482 (26.8) | <0.01 |
| PTSD only | 499 (38.8) | 593 (42.3) | 371 (41.8) | 27 (40.3) | 0.30 | 730 (38.5) | 765 (42.5) | <0.05 |
| **Symptoms of two conditions** |  |  |  |  |  |  |  |  |
| Depression and anxiety | 125 (9.7) | 153 (10.9) | 114 (12.8) | 12 (17.9) | <0.05 | 177 (9.3) | 224 (12.4) | <0.01 |
| Depression and PTSD | 146 (11.4) | 184 (13.1) | 104 (11.7) | 11 (16.4) | 0.35 | 207 (10.9) | 237 (13.2) | <0.05 |
| Anxiety and PTSD | 198 (15.4) | 254 (18.1) | 151 (17.0) | 12 (17.9) | 0.31 | 279 (14.7) | 340 (18.9) | <0.01 |
| **Symptoms of three conditions** |  |  |  |  |  |  |  |  |
| Depression, anxiety, and PTSD | 96 (7.5) | 130 (9.3) | 81 (9.1) | 8 (11.9) | 0.24 | 146 (7.7) | 166 (9.2) | 0.09 |

<https://doi.org/10.1207/s15327906mbr2603_7>

Don't worry; you already have enough participants.

We understand the importance of establishing an adequate sample size, especially when conducting multiple regression analyses, to ensure statistical power and reliability in our findings. We have taken this into account in our study and carefully considered the need for an appropriate number of respondents to support our analyses.

Regarding the provided reference, we have reviewed the paper and its recommendations regarding the determination of the minimum sample size for regression analyses. We agree that having sufficient participants is essential for the robustness of our analysis.

We assure you that we have carefully considered the statistical requirements and have taken steps to ensure that our study has an appropriate number of participants to support our multiple regression analyses and allow us to draw meaningful conclusions from the data.

2. In Table 3, please report the exact p-value

**Standard Reporting of P-Values:**

SPSS, like other statistical software, typically reports p-values to three decimal places. However, it is important to note that this level of precision does not imply a higher level of significance. P-values below 0.05 are often reported as "< 0.001" or "< .001" for practical reasons related to formatting and readability.

Reviewer #5: Thank you for addressing most of the comments. I have a few minor edit suggestion:

1. Abstract, "For gender differences, symptoms of the three conditions were higher in female students (9.2%) compared to male peers (7.7%) (p<0.05)." Three conditions were higher in the female, but only one % is reported. This percentage represents which condition?

Done! For gender differences, symptoms of the three conditions (Depression, anxiety, and PTSD) were higher in female students (9.2%) compared to male peers (7.7%) (p<0.05).

2. "similarly, witnessing the death of a close family member due to COVID-19 (OR=1.7, p<0.01) were positive predictors associated with PTDS, depression, and anxiety." This OR represents which conditions?
Done! The OR represents all three conditions (PTSD, depression, and anxiety)

3. In the statistical analysis section, the authors are suggested to report how the variables were selected for multivariable logistics regression model.

In the variable selection process for the multivariable logistic regression model, we carefully considered several factors to ensure a robust and appropriate selection of variables that are relevant to our research question. These considerations were based on both statistical and substantive reasoning.

Relevance to Research Question:

We assessed the theoretical relevance of each variable to the research question and objectives of our study. Variables directly related to the phenomenon under investigation were given higher priority.

Literature Review:

A comprehensive literature review guided the selection of variables. We incorporated findings from previous research and existing theoretical frameworks to identify variables with established associations with the outcomes of interest.

Clinical Expertise:

We consulted with domain experts, including clinicians and subject matter experts, to ensure that the variables selected align with clinical knowledge and expertise related to the research topic.

Statistical Significance:

Statistical significance of variables was evaluated using appropriate tests, such as chi-square tests o, depending on the nature of the variables (categorical or continuous). Variables with a significant association (p-value below a pre-defined threshold, e.g., p < 0.05) were considered for inclusion.

4. In my earlier revision, I suggested the authors to expand the discussion section discussing all the significant variables and also suggested a few articles for their help. However, the authors did not elaborate the discussion section.
We have added the suggested articles in the discussion section:

Chowdhury SR, Sunna TC, Das DC, Kabir H, Hossain A, Mahmud S, Ahmed S. Mental health symptoms among the nurses of Bangladesh during the COVID-19 pandemic. Middle East Curr Psychiatry. 2021;28(1):23. doi: 10.1186/s43045-021-00103-x.

Chowdhury SR, Kabir H, Mazumder S, Akter N, Chowdhury MR, Hossain A. Workplace violence, bullying, burnout, job satisfaction and their correlation with depression among Bangladeshi nurses: A cross-sectional survey during the COVID-19 pandemic. PLoS One. 2022 Sep 22;17(9):e0274965. doi: 10.1371/journal.pone.0274965.
