# Supplementary material for: Prevalence and factors associated with mental illness symptoms among school students post lockdown of the COVID-19 pandemic in the United Arab Emirates: A cross-sectional national study
Source: PLoS One. 2024 Feb 1;19(2):e0296479. doi: 10.1371/journal.pone.0296479 (PMC10833540; doi:10.1371/journal.pone.0296479)
Supplement: Attachment — Submitted filename: Response to reviewers(Revised-Nov23-2023).docx [file pone.0296479.s004.docx]

Dear Reviewers,

Thank you again for your valuable feedback. Please find below the modifications we introduced based on your comments.

Regards,

6. Review Comments to the Author

Reviewer #1: We thank the authors for addressing our comments in tge previous version and the following are our concern in this version ;

1. It fails to present whether descriptive or analytic study and the rationale behind

The study uses the descriptive study design (please check lines 136-142).

Rationale:

Literature affirms that descriptive studies are best used to describe the distribution of one or more variables, devoid of causal links. Furthermore, this type of studies is considered instrumental in measuring the prevalence of a disease or of a risk factor in a population (Aggarawal & Ranganathan, 2019). In this study, the focus is on discerning the prevalence and factors associated with mental illness symptoms among school students in the COVID-19 post-lockdown phase. Recognizing the efficacy of descriptive studies in unveiling patterns and trends, researchers anticipate that this design will uncover commonalities, variations, or trends among students, offering invaluable insights for targeted interventions in the realm of mental health symptoms.

Aggarwal R, Ranganathan P. Study designs: Part 2 - Descriptive studies. Perspect Clin Res. 2019 Jan-Mar;10(1):34-36. doi: 10.4103/picr.PICR_154_18. PMID: 30834206; PMCID: PMC6371702.
2.use of present tense e.g 'are' in the abstract section .

Done (please check lines no. 35-36)

3.covinience and snow ball sampling for quantitative study is unusual.

Due to the limitations imposed by the COVID-19 pandemic, many researchers on similar studies (please see references listed below) used the convenience and snowball sampling techniques (Abdelrahman, 2022; Cheikh, 2021; Vajpeyi, 2022). More precisely, this technique was adopted by researchers in the UAE due to the inherent interconnectedness of the population under study. Leveraging existing networks and connections within the target population facilitated access to individuals who may have otherwise been challenging to reach through traditional sampling methods. We acknowledge the limitations associated with convenience and snowball sampling and we have provided a thorough discussion of these limitations in the revised manuscript.

References:

1. Abdelrahman R, Ismail ME. The psychological distress and covid-19 pandemic during lockdown: A cross-sectional study from United Arab Emirates (UAE). Heliyon. 2022;8(5). doi:10.1016/j.heliyon.2022.e09422
2. Cheikh Ismail L, Mohamad MN, Bataineh MF, Ajab A, Al-Marzouqi AM, Jarrar AH, et al. Impact of the coronavirus pandemic (COVID-19) lockdown on mental health and well-being in the United Arab Emirates. Frontiers in Psychiatry. 2021;12. doi:10.3389/fpsyt.2021.633230
3. Vajpeyi Misra A, Mamdouh HM, Dani A, Mitchell V, Hussain HY, Ibrahim GM, et al. Impact of covid-19 pandemic on the mental health of university students in the United Arab Emirates: A cross-sectional study. BMC Psychology. 2022;10(1). doi:10.1186/s40359-022-00986-3

4.The background fails to cite the global and national initiatives promised to prevent and control mental health and its symptoms

More global and national initiatives were added (please check lines 80-87).

5.The data quality assurance is missed e.g is all the collected data complete ?

Data quality assurance was added (please check lines 190-203).

6. Ethics issues:
_Age of respondents and the way consent was secured

Age of respondents ranged from 8-18 years (please check lines 148-149).

Students were requested to confirm their consent by responding to one mandatory written question just before they answered the electronic survey questions. Participants were informed they could stop completing the questionnaire or refrain from submitting their responses if they felt uncomfortable at any stage during completion. Participation in the study was voluntary. Participants had the full right to withdraw at any time without the need to justify their actions. (Please check lines 239-243)

_What was done for those with mental health illness:

Considering that students with special needs were excluded from the study, all students who met the inclusion criteria were included in data analysis.

_Is there funding?If so the role of funder should be stated.

The study did not receive any funding. However, the publication fees will be covered by the Emirates Health Services which is recognized in the acknowledgement section.

_Authors contributions is not described

Authors’ contributions followed the universal requirement for authorship as well as those of PLOS ONE.

<https://hms.harvard.edu/sites/default/files/assets/Sites/Ombuds/files/AUTHORSHIP%20GUIDELINES.pdf>

7.The tables are many /5/.Present some of the tables in narration form and it lacks self explanation. In addition usw figurea for most attracting findings.

To address your concern about self-explanation, we enhanced the narrative descriptions accompanying the tables to ensure a comprehensive and clear understanding of the presented data. Furthermore, we have added one figure.

As for the number of tables, we believe all of them are useful for presenting the multiple datasets in an organized manner.

8. The discussion is not in line with the guidelines of the journal.

We again revisited the guidelines of the journal as shown below. One suggestion may be to merge the discussion and conclusion parts under one section labeled “Discussion”. Perhaps the editor can guide us in this regard.

<https://journals.plos.org/plosone/s/submission-guidelines>

9 Statistics
-The percentage are not exactly 100%

-Use confidence interval while presenting percentage

We would like to clarify that the discrepancy in percentages is attributed to inapplicable/unanswered responses in the dataset. Some participants chose not to respond to certain items, resulting in a non-valid dataset for certain variables. If you add these responses together it equals 100%. We acknowledge the importance of transparency in reporting and will explicitly mention in the revised manuscript that the percentages presented are based on the number of valid responses for each item.

We appreciate your suggestion regarding the use of confidence intervals to present percentages in our study. While we acknowledge the utility of confidence intervals in many statistical contexts, it is important to note that presenting confidence intervals for percentages is not a common practice in certain fields, particularly in reporting prevalence. Percentages are typically reported as point estimates, representing the proportion of a sample that exhibits a particular characteristic or response. Confidence intervals are more commonly used for continuous variables or when estimating parameters of a population distribution. We have already presented confidence intervals in Table 5. Applying confidence intervals to percentages may lead to misinterpretations, as they are inherently bounded between 0% and 100%, and confidence intervals could extend beyond these limits. To enhance clarity and adherence to standard reporting practices in our field, we have chosen to present percentages as point estimates in the manuscript.

Reviewer #4: Thank you for addressing the previous comments; the manuscript is ready for publication.

On a side note, it is essential to emphasize that reporting the exact P values is crucial in research. It is essential to note that not all p-values below 0.05 should be reported as <0.001, however, when the software output displayed the p-value as "000," the researchers reported it as <0.001.

Thank you for the side note, we have already reported the exact p values in the last revision. We appreciate your attention to detail, particularly regarding the reporting of P values. We acknowledge the importance of accurate and transparent reporting in research. There were specific instances where the software output displayed a P-value as "<0.001,"and so we reported the same in the revised version and we understand the necessity of accurately representing such values rather than automatically reporting them.

Reviewer #5: Thanks for addressing most of the comments. However, the manuscript still requires improvement to get it published in a high-quality journal.

A few comments below:

1. Discuss elaborately the tools used for assessing three psychological conditions. Items of the tools, their cut-off values, and the cut-off used to determine the presence of three conditions. The scales' reliability and validity in this population.

Tools are elaborated on page number 7.

Cut off is mentioned on page 14

2. Discuss in the statistical analysis section how the variables were selected for multivariable analysis.

we have enhanced the statistical analysis section to provide a detailed discussion on the criteria and rationale behind the selection of variables for the multivariable analysis. This included a thorough explanation of the factors considered, such as variable significance, relevance to the research question, and pre-established criteria for inclusion. By addressing this aspect more explicitly, we aimed at offering readers a clearer understanding of the methodological choices made during the analysis.

3. Avoid using the informal short form in the result section (eg. yrs).

Done

4. The presentation in the table is messy and difficult to understand. Improve the presentation of the data in the tables. You can make the cell borders visible to make the table clear.

The formatting of the table is done as requested.

Thanks.
