# Supplementary material for: Prevalence and factors associated with mental illness symptoms among school students post lockdown of the COVID-19 pandemic in the United Arab Emirates: A cross-sectional national study
Source: PLoS One. 2024 Feb 1;19(2):e0296479. doi: 10.1371/journal.pone.0296479 (PMC10833540; doi:10.1371/journal.pone.0296479)
Supplement: Peer review history

# Peer review: Decision Letter 0

**DOI:** 10.1371/journal.pone.0296479.r001

**Authors:** Humayun Kabir

**Transfer Alert**

This paper was transferred from another journal. As a result, its full editorial history (including decision letters, peer reviews and author responses) may not be present.

27 Dec 2022

PONE-D-22-19659Prevalence and Factors Associated with Mental Illness Symptoms among School Students Post Lockdown of the COVID-19 Pandemic in the United Arab Emirates: A Cross-Sectional National StudyPLOS ONE

Dear Dr. Ghader,

Thank you for submitting your manuscript to PLOS ONE. After careful consideration, we feel that it has merit but does not fully meet PLOS ONE’s publication criteria as it currently stands. Therefore, we invite you to submit a revised version of the manuscript that addresses the points raised during the review process.

Please submit your revised manuscript by January 30, 2023. If you will need more time than this to complete your revisions, please reply to this message or contact the journal office at plosone@plos.org. When you're ready to submit your revision, log on to https://www.editorialmanager.com/pone/ and select the 'Submissions Needing Revision' folder to locate your manuscript file.

Please include the following items when submitting your revised manuscript:

- A rebuttal letter that responds to each point raised by the academic editor and reviewer(s). You should upload this letter as a separate file labeled 'Response to Reviewers'.
- A marked-up copy of your manuscript that highlights changes made to the original version. You should upload this as a separate file labeled 'Revised Manuscript with Track Changes'.
- An unmarked version of your revised paper without tracked changes. You should upload this as a separate file labeled 'Manuscript'.

If you would like to make changes to your financial disclosure, please include your updated statement in your cover letter. Guidelines for resubmitting your figure files are available below the reviewer comments at the end of this letter.

If applicable, we recommend that you deposit your laboratory protocols in protocols.io to enhance the reproducibility of your results. Protocols.io assigns your protocol its own identifier (DOI) so that it can be cited independently in the future. For instructions see: https://journals.plos.org/plosone/s/submission-guidelines#loc-laboratory-protocols. Additionally, PLOS ONE offers an option for publishing peer-reviewed Lab Protocol articles, which describe protocols hosted on protocols.io. Read more information on sharing protocols at https://plos.org/protocols?utm_medium=editorial-email&utm_source=authorletters&utm_campaign=protocols.

We look forward to receiving your revised manuscript.

Kind regards,

Humayun Kabir, MSc in Epidemiology

Academic Editor

PLOS ONE

Journal Requirements:

When submitting your revision, we need you to address these additional requirements.

1. Please ensure that your manuscript meets PLOS ONE's style requirements, including those for file naming. The PLOS ONE style templates can be found at

https://journals.plos.org/plosone/s/file?id=wjVg/PLOSOne_formatting_sample_main_body.pdf and

https://journals.plos.org/plosone/s/file?id=ba62/PLOSOne_formatting_sample_title_authors_affiliations.pdf

2. Please provide additional details regarding participant consent. In the ethics statement in the Methods and online submission information, please ensure that you have specified what type you obtained (for instance, written or verbal, and if verbal, how it was documented and witnessed). If your study included minors, state whether you obtained consent from parents or guardians. If the need for consent was waived by the ethics committee, please include this information.

3. We note that you have indicated that data from this study are available upon request. PLOS only allows data to be available upon request if there are legal or ethical restrictions on sharing data publicly. For more information on unacceptable data access restrictions, please see http://journals.plos.org/plosone/s/data-availability#loc-unacceptable-data-access-restrictions.

In your revised cover letter, please address the following prompts:

a) If there are ethical or legal restrictions on sharing a de-identified data set, please explain them in detail (e.g., data contain potentially sensitive information, data are owned by a third-party organization, etc.) and who has imposed them (e.g., an ethics committee). Please also provide contact information for a data access committee, ethics committee, or other institutional body to which data requests may be sent.

b) If there are no restrictions, please upload the minimal anonymized data set necessary to replicate your study findings as either Supporting Information files or to a stable, public repository and provide us with the relevant URLs, DOIs, or accession numbers. For a list of acceptable repositories, please see http://journals.plos.org/plosone/s/data-availability#loc-recommended-repositories.

We will update your Data Availability statement on your behalf to reflect the information you provide.

[Note: HTML markup is below. Please do not edit.]

Reviewers' comments:

Reviewer's Responses to Questions

**Comments to the Author**

1. Is the manuscript technically sound, and do the data support the conclusions?

The manuscript must describe a technically sound piece of scientific research with data that supports the conclusions. Experiments must have been conducted rigorously, with appropriate controls, replication, and sample sizes. The conclusions must be drawn appropriately based on the data presented. 

Reviewer #1: Yes

Reviewer #2: Yes

**********

2. Has the statistical analysis been performed appropriately and rigorously? 

Reviewer #1: Yes

Reviewer #2: I Don't Know

**********

3. Have the authors made all data underlying the findings in their manuscript fully available?

The PLOS Data policy requires authors to make all data underlying the findings described in their manuscript fully available without restriction, with rare exception (please refer to the Data Availability Statement in the manuscript PDF file). The data should be provided as part of the manuscript or its supporting information, or deposited to a public repository. For example, in addition to summary statistics, the data points behind means, medians and variance measures should be available. If there are restrictions on publicly sharing data—e.g. participant privacy or use of data from a third party—those must be specified.

Reviewer #1: Yes

Reviewer #2: Yes

**********

4. Is the manuscript presented in an intelligible fashion and written in standard English?

PLOS ONE does not copyedit accepted manuscripts, so the language in submitted articles must be clear, correct, and unambiguous. Any typographical or grammatical errors should be corrected at revision, so please note any specific errors here.

Reviewer #1: Yes

Reviewer #2: Yes

**********

5. Review Comments to the Author

Please use the space provided to explain your answers to the questions above. You may also include additional comments for the author, including concerns about dual publication, research ethics, or publication ethics. (Please upload your review as an attachment if it exceeds 20,000 characters)

Reviewer #1: Dear PLOS ONE team of Editorials, thank you for the chance given to me to re-review “Deworming utilization among pregnant mothers with at least one Antenatal care follow up in Ethiopia, 2022: - A Multilevel analysis” with manuscript Number PONE-D-22-26022R1. I am thankful for addressing and correcting the [previous version of the manuscript. The following are my comments in this version;

1. In your response to my comment regarding regional deworming status you have responded as “Hence, we did not show it in the multivariable model t. Nevertheless, the highest prevalence is observed in Dire Dewa town (13.8%) and the lowest one in Somali region (4.4%)”. Therefore, try to also incorporate this in the manuscript as well since this is also one of the findings.

2. The mass campaign deworming Vs your response “ , those mothers with poor wealth index/ economic status will not routinely utilize health services due to various reasons like transportation issues, their poor knowledge regarding to such services etc. If the pregnant mother does seek health institutions regularly, she will not have a chance to get deworming and other complementary ANC services.” are contraindicating and have you analyzed it specifically? Otherwise, you were cross-reasoning like …poor poverty…NOT have chance to get deworming and OTHER?

3. Try to ensure all the sections for their main and key contents.

4. Reference your classifications well e.g., educational level (High and Low), Income…etc.

5. Avoid repetition and maintain consistency particularly on scientific rigor.

6. Minor language, full stop missing, capital letters and abbreviations, and statistical corrections are needed e.g. Moslem to mean Muslim and Addis Abeba to mean Addis Ababa, catholic instead of Catholic, statistics not beginning from higher to lower.

7. Meticulous proof read is needed before the actual publication.

Reviewer #2: Thank you for inviting me to review this article. This is a study on the prevalence of mental health symptoms and its associated factors among a large sample of children and adolescents in the UAE. I enjoyed reading the article and have several suggestions to improve it.

There were contradictory statements regarding the data availaibility. ‘Yes - all data are fully available without restriction’, / Data are available from the Emirates Health Services Institutional Data Access / Ethics Committee (contact via MHD@ehs.gov.ae) for researchers who meet the criteria for access to confidential data.

INTRODUCTION

Paragraph 2

There has been lack of reference to the current state of evidence regarding the impact of the pandemic on the mental health of children and young people. What has systematic reviews/meta-analyses within this topic has found? I suggest for authors to conduct a simple literature search to find relevant reviews on this topic.

Search string that can be used e.g ‘systematic review’ AND ‘mental health’ AND COVID-19 AND (‘young people’ OR children OR adolescent) and you can find several related reviews

Some recently published examples that might be relevant for consideration:

Mental health changes before vs during the pandemic: https://doi.org/10.1007/s00787-022-02060-0

Impact on clinical groups: https://doi.org/10.1177/20503121221086712

Prevalence and correlates of mental health: https://doi.org/10.1080/23311908.2022.2111849

Paragraph 3-4

There appears to be a number of related studies in UAE already so I suggest a brief sentence/sentences that highlight how this current study build on these already published works.

METHODS

Can you clarify the total population of the students and sampling strategy used? While it is clear n=xx were eventually recruited, how many participants were invited? It seems that all private and public schools in UAE were invited therefore it might be possible to obtain the numbers and estimate total population and how the sample was obtained.

I notice the authors define the age groups into; Pre-adolescence (<10 years - ?what’s the minimum age), Early adolescence (10-13 years), Middle adolescence (14-17 years) and Late adolescence (18 year). There has been variations of age groups definition and terminologies for children, adolescent and young people etc. Can you share any reference or justification for the age group division that you used? Are these age groups relate to the grades/schooling system in the UAE?

RESULTS

It was clarified that for table 1 ‘Percentages may not add up to 100 because of missing data’. I suggest the descriptive amount and percentage of missing data to be included in the table.

STRENGTHS AND LIMITATIONS

There was mentioned about a pilot test being done which i did not find earlier. I suggest to describe the details of the pilot test in Methods. Worth noting that the study is observational therefore unable to make causation of the pandemic > mental health, and cross-sectional so unable to ascertain whether it got worse than before pandemic. It was also a bit unclear whether the large sample size can be generalized to the nationwide population level, given unclear if it uses probability vs nonprobability sampling method.

**********

6. PLOS authors have the option to publish the peer review history of their article (what does this mean?). If published, this will include your full peer review and any attached files.

If you choose “no”, your identity will remain anonymous but your review may still be made public.

**Do you want your identity to be public for this peer review?** For information about this choice, including consent withdrawal, please see our Privacy Policy.

Reviewer #1: No

Reviewer #2: No

**********

[NOTE: If reviewer comments were submitted as an attachment file, they will be attached to this email and accessible via the submission site. Please log into your account, locate the manuscript record, and check for the action link "View Attachments". If this link does not appear, there are no attachment files.]

While revising your submission, please upload your figure files to the Preflight Analysis and Conversion Engine (PACE) digital diagnostic tool, https://pacev2.apexcovantage.com/. PACE helps ensure that figures meet PLOS requirements. To use PACE, you must first register as a user. Registration is free. Then, login and navigate to the UPLOAD tab, where you will find detailed instructions on how to use the tool. If you encounter any issues or have any questions when using PACE, please email PLOS at figures@plos.org. Please note that Supporting Information files do not need this step.

<quillbot-extension-portal></quillbot-extension-portal><quillbot-extension-portal></quillbot-extension-portal>

# Author comment: Author response to Decision Letter 0

**DOI:** 10.1371/journal.pone.0296479.r002

8 Mar 2023

Prevalence and Factors Associated with Mental Illness Symptoms among School Students Post Lockdown of the COVID-19 Pandemic in the United Arab Emirates: A Cross-Sectional National Study 

Dear Reviewers

On behalf of the research team, we would like to thank you for the very valuable feedback. Your suggestions are developmental and will definitely make our work immaculate. Please find our responses marked in olive green.

Journal Requirements:

When submitting your revision, we need you to address these additional requirements.

1. Please ensure that your manuscript meets PLOS ONE's style requirements, including those for file naming. The PLOS ONE style templates can be found at

https://journals.plos.org/plosone/s/file?id=wjVg/PLOSOne_formatting_sample_main_body.pdf and

https://journals.plos.org/plosone/s/file?id=ba62/PLOSOne_formatting_sample_title_authors_affiliations.pdf

The manuscript and other relevant documents were prepared per PLOS ONE's style requirements. 

2. Please provide additional details regarding participant consent. In the ethics statement in the Methods and online submission information, please ensure that you have specified what type you obtained (for instance, written or verbal, and if verbal, how it was documented and witnessed). If your study included minors, state whether you obtained consent from parents or guardians. If the need for consent was waived by the ethics committee, please include this information.

Details on participant consent were added. 

• They can be tracked in the manuscript (lines 146-147, 169-172). 

• For the online submission information (online questionnaire) , details are found on the Participant Information Sheet, section “Participants’ role in the study”, sub-section “Part 1 (Questions 1-11)” 

3. We note that you have indicated that data from this study are available upon request. PLOS only allows data to be available upon request if there are legal or ethical restrictions on sharing data publicly. For more information on unacceptable data access restrictions, please see http://journals.plos.org/plosone/s/data-availability#loc-unacceptable-data-access-restrictions.

In your revised cover letter, please address the following prompts:

a) If there are ethical or legal restrictions on sharing a de-identified data set, please explain them in detail (e.g., data contain potentially sensitive information, data are owned by a third-party organization, etc.) and who has imposed them (e.g., an ethics committee). Please also provide contact information for a data access committee, ethics committee, or other institutional body to which data requests may be sent.

b) If there are no restrictions, please upload the minimal anonymized data set necessary to replicate your study findings as either Supporting Information files or to a stable, public repository and provide us with the relevant URLs, DOIs, or accession numbers. For a list of acceptable repositories, please see http://journals.plos.org/plosone/s/data-availability#loc-recommended-repositories.

The below statement has been added to the revised cover letter:

“Anonymized data set necessary to replicate our study findings is made available on a public data repository (Figshare) 

https://doi.org/10.6084/m9.figshare.22231585)

The below statement was added to the manuscript (Lines 157-159). Reference (Ref # 17)

“Anonymized data set necessary to replicate the study findings is available to other researchers on a public repository [17].”

We will update your Data Availability statement on your behalf to reflect the information you provide.

[Note: HTML markup is below. Please do not edit.]

Reviewers' comments:

Reviewer's Responses to Questions

Comments to the Author

1. Is the manuscript technically sound, and do the data support the conclusions?

The manuscript must describe a technically sound piece of scientific research with data that supports the conclusions. Experiments must have been conducted rigorously, with appropriate controls, replication, and sample sizes. The conclusions must be drawn appropriately based on the data presented. 

Reviewer #1: Yes

Reviewer #2: Yes

________________________________________

2. Has the statistical analysis been performed appropriately and rigorously? 

Reviewer #1: Yes

Reviewer #2: I Don't Know

Data set is now accessible to reviewer for evaluation.

________________________________________

3. Have the authors made all data underlying the findings in their manuscript fully available?

The PLOS Data policy requires authors to make all data underlying the findings described in their manuscript fully available without restriction, with rare exception (please refer to the Data Availability Statement in the manuscript PDF file). The data should be provided as part of the manuscript or its supporting information, or deposited to a public repository. For example, in addition to summary statistics, the data points behind means, medians and variance measures should be available. If there are restrictions on publicly sharing data—e.g. participant privacy or use of data from a third party—those must be specified.

Reviewer #1: Yes

Reviewer #2: Yes

________________________________________

4. Is the manuscript presented in an intelligible fashion and written in standard English?

PLOS ONE does not copyedit accepted manuscripts, so the language in submitted articles must be clear, correct, and unambiguous. Any typographical or grammatical errors should be corrected at revision, so please note any specific errors here.

Reviewer #1: Yes

Reviewer #2: Yes

________________________________________

5. Review Comments to the Author

Please use the space provided to explain your answers to the questions above. You may also include additional comments for the author, including concerns about dual publication, research ethics, or publication ethics. (Please upload your review as an attachment if it exceeds 20,000 characters)

Reviewer #1: Dear PLOS ONE team of Editorials, thank you for the chance given to me to re-review “Deworming utilization among pregnant mothers with at least one Antenatal care follow up in Ethiopia, 2022: - A Multilevel analysis” with manuscript Number PONE-D-22-26022R1. I am thankful for addressing and correcting the [previous version of the manuscript. The following are my comments in this version;

1. In your response to my comment regarding regional deworming status you have responded as “Hence, we did not show it in the multivariable model t. Nevertheless, the highest prevalence is observed in Dire Dewa town (13.8%) and the lowest one in Somali region (4.4%)”. Therefore, try to also incorporate this in the manuscript as well since this is also one of the findings.

2. The mass campaign deworming Vs your response “ , those mothers with poor wealth index/ economic status will not routinely utilize health services due to various reasons like transportation issues, their poor knowledge regarding to such services etc. If the pregnant mother does seek health institutions regularly, she will not have a chance to get deworming and other complementary ANC services.” are contraindicating and have you analyzed it specifically? Otherwise, you were cross-reasoning like …poor poverty…NOT have chance to get deworming and OTHER?

3. Try to ensure all the sections for their main and key contents.

4. Reference your classifications well e.g., educational level (High and Low), Income…etc.

5. Avoid repetition and maintain consistency particularly on scientific rigor.

6. Minor language, full stop missing, capital letters and abbreviations, and statistical corrections are needed e.g. Moslem to mean Muslim and Addis Abeba to mean Addis Ababa, catholic instead of Catholic, statistics not beginning from higher to lower.

7. Meticulous proof read is needed before the actual publication.

This review is irrelevant to our study.

Reviewer #2: Thank you for inviting me to review this article. This is a study on the prevalence of mental health symptoms and its associated factors among a large sample of children and adolescents in the UAE. I enjoyed reading the article and have several suggestions to improve it.

There were contradictory statements regarding the data availaibility. ‘Yes - all data are fully available without restriction’, / Data are available from the Emirates Health Services Institutional Data Access / Ethics Committee (contact via MHD@ehs.gov.ae) for researchers who meet the criteria for access to confidential data.

Data availability was corrected in the cover letter and the manuscript (Lines 157-159) 

INTRODUCTION

Paragraph 2

There has been lack of reference to the current state of evidence regarding the impact of the pandemic on the mental health of children and young people. What has systematic reviews/meta-analyses within this topic has found? I suggest for authors to conduct a simple literature search to find relevant reviews on this topic.

Search string that can be used e.g ‘systematic review’ AND ‘mental health’ AND COVID-19 AND (‘young people’ OR children OR adolescent) and you can find several related reviews

Some recently published examples that might be relevant for consideration:

Mental health changes before vs during the pandemic: https://doi.org/10.1007/s00787-022-02060-0

Impact on clinical groups: https://doi.org/10.1177/20503121221086712

Prevalence and correlates of mental health: https://doi.org/10.1080/23311908.2022.2111849

Data from a systematic review were added (lines 58-62). Reference (Ref # 4) 

Paragraph 3-4

There appears to be a number of related studies in UAE already so I suggest a brief sentence/sentences that highlight how this current study build on these already published works.

Two studies from the UAE were added (lines 92-96). References (Ref # 10 and 11) 

METHODS

Can you clarify the total population of the students and sampling strategy used? While it is clear n=xx were eventually recruited, how many participants were invited? It seems that all private and public schools in UAE were invited therefore it might be possible to obtain the numbers and estimate total population and how the sample was obtained.

The invite was sent out to all students in private and public schools aged 8-18. The total population has been estimated at about 721048 per the UAE Open Data Portal https://data.government.ae/dataset?tags=ODT&tags=education&res_format=XLSX&organization=ministry-of-education. Total population sampling was used to obtain the sample. 

Please note that the above explanation was added in the manuscript (lines 127-129). Reference (Ref # 12) 

I notice the authors define the age groups into; Pre-adolescence (<10 years - ?what’s the minimum age) minimum age is 8, Early adolescence (10-13 years), Middle adolescence (14-17 years) and Late adolescence (18 year). There has been variations of age groups definition and terminologies for children, adolescent and young people etc. Can you share any reference or justification for the age group division that you used? Are these age groups relate to the grades/schooling system in the UAE?

Age groups were identified based on two resources: 

1. the UAE stages of school education

Stages and streams of school education - the official portal of the UAE Government. Retrieved March 6, 2023, from https://u.ae/en/information-and-services/education/school-education-k-12/joining-k-12-education/stages-and-streams-of-school-education

2. Published Article:

Salmela-Aro, K. (2011). Stages of adolescence. Encyclopedia of Adolescence, 360–368. https://doi.org/10.1016/b978-0-12-373951-3.00043-0

A note on the age group was added to the manuscript (Lines 198-199). References (Ref # 18 and 19) 

RESULTS

It was clarified that for table 1 ‘Percentages may not add up to 100 because of missing data’. I suggest the descriptive amount and percentage of missing data to be included in the table.

Data on table 1 (total and missing entries) were added. 

STRENGTHS AND LIMITATIONS

There was mentioned about a pilot test being done which i did not find earlier. I suggest to describe the details of the pilot test in Methods. Worth noting that the study is observational therefore unable to make causation of the pandemic > mental health, and cross-sectional so unable to ascertain whether it got worse than before pandemic. It was also a bit unclear whether the large sample size can be generalized to the nationwide population level, given unclear if it uses probability vs nonprobability sampling method.

Pilot test was added to the method section (Lines 152-153).

________________________________________

6. PLOS authors have the option to publish the peer review history of their article (what does this mean?). If published, this will include your full peer review and any attached files.

If you choose “no”, your identity will remain anonymous but your review may still be made public.

Do you want your identity to be public for this peer review? For information about this choice, including consent withdrawal, please see our Privacy Policy.

Reviewer #1: No

Reviewer #2: No

Attachment Submitted filename: Response to Reviewers.docx Click here for additional data file.

# Peer review: Decision Letter 1

**DOI:** 10.1371/journal.pone.0296479.r003

**Authors:** Humayun Kabir

21 Jul 2023

PONE-D-22-19659R1Prevalence and Factors Associated with Mental Illness Symptoms among School Students Post Lockdown of the COVID-19 Pandemic in the United Arab Emirates: A Cross-Sectional National StudyPLOS ONE

Dear Dr. Ghader,

Thank you for submitting your manuscript to PLOS ONE. After careful consideration, we feel that it has merit but does not fully meet PLOS ONE’s publication criteria as it currently stands. Therefore, we invite you to submit a revised version of the manuscript that addresses the points raised during the review process.

Please submit your revised manuscript by August 20, 2023. If you will need more time than this to complete your revisions, please reply to this message or contact the journal office at plosone@plos.org. When you're ready to submit your revision, log on to https://www.editorialmanager.com/pone/ and select the 'Submissions Needing Revision' folder to locate your manuscript file.

Please include the following items when submitting your revised manuscript:

- A rebuttal letter that responds to each point raised by the academic editor and reviewer(s). You should upload this letter as a separate file labeled 'Response to Reviewers'.
- A marked-up copy of your manuscript that highlights changes made to the original version. You should upload this as a separate file labeled 'Revised Manuscript with Track Changes'.
- An unmarked version of your revised paper without tracked changes. You should upload this as a separate file labeled 'Manuscript'.

If you would like to make changes to your financial disclosure, please include your updated statement in your cover letter. Guidelines for resubmitting your figure files are available below the reviewer comments at the end of this letter.

If applicable, we recommend that you deposit your laboratory protocols in protocols.io to enhance the reproducibility of your results. Protocols.io assigns your protocol its own identifier (DOI) so that it can be cited independently in the future. For instructions see: https://journals.plos.org/plosone/s/submission-guidelines#loc-laboratory-protocols. Additionally, PLOS ONE offers an option for publishing peer-reviewed Lab Protocol articles, which describe protocols hosted on protocols.io. Read more information on sharing protocols at https://plos.org/protocols?utm_medium=editorial-email&utm_source=authorletters&utm_campaign=protocols.

We look forward to receiving your revised manuscript.

Kind regards,

Humayun Kabir

Academic Editor

PLOS ONE

Journal Requirements:

Please review your reference list to ensure that it is complete and correct. If you have cited papers that have been retracted, please include the rationale for doing so in the manuscript text, or remove these references and replace them with relevant current references. Any changes to the reference list should be mentioned in the rebuttal letter that accompanies your revised manuscript. If you need to cite a retracted article, indicate the article’s retracted status in the References list and also include a citation and full reference for the retraction notice.

Additional Editor Comments (if provided):

Dear Author, reviewers still have some concerns as a mix of major and minor comments. Could you please try to address and return the manuscript with your revise version? Thank you!

[Note: HTML markup is below. Please do not edit.]

Reviewers' comments:

Reviewer's Responses to Questions

**Comments to the Author**

1. If the authors have adequately addressed your comments raised in a previous round of review and you feel that this manuscript is now acceptable for publication, you may indicate that here to bypass the “Comments to the Author” section, enter your conflict of interest statement in the “Confidential to Editor” section, and submit your "Accept" recommendation.

Reviewer #1: All comments have been addressed

Reviewer #3: (No Response)

Reviewer #4: (No Response)

Reviewer #5: (No Response)

**********

2. Is the manuscript technically sound, and do the data support the conclusions?

The manuscript must describe a technically sound piece of scientific research with data that supports the conclusions. Experiments must have been conducted rigorously, with appropriate controls, replication, and sample sizes. The conclusions must be drawn appropriately based on the data presented. 

Reviewer #1: No

Reviewer #3: Yes

Reviewer #4: Partly

Reviewer #5: Partly

**********

3. Has the statistical analysis been performed appropriately and rigorously? 

Reviewer #1: No

Reviewer #3: Yes

Reviewer #4: Yes

Reviewer #5: No

**********

4. Have the authors made all data underlying the findings in their manuscript fully available?

The PLOS Data policy requires authors to make all data underlying the findings described in their manuscript fully available without restriction, with rare exception (please refer to the Data Availability Statement in the manuscript PDF file). The data should be provided as part of the manuscript or its supporting information, or deposited to a public repository. For example, in addition to summary statistics, the data points behind means, medians and variance measures should be available. If there are restrictions on publicly sharing data—e.g. participant privacy or use of data from a third party—those must be specified.

Reviewer #1: Yes

Reviewer #3: Yes

Reviewer #4: Yes

Reviewer #5: Yes

**********

5. Is the manuscript presented in an intelligible fashion and written in standard English?

PLOS ONE does not copyedit accepted manuscripts, so the language in submitted articles must be clear, correct, and unambiguous. Any typographical or grammatical errors should be corrected at revision, so please note any specific errors here.

Reviewer #1: Yes

Reviewer #3: No

Reviewer #4: Yes

Reviewer #5: Yes

**********

6. Review Comments to the Author

Please use the space provided to explain your answers to the questions above. You may also include additional comments for the author, including concerns about dual publication, research ethics, or publication ethics. (Please upload your review as an attachment if it exceeds 20,000 characters)

Reviewer #1: Review Report

Title: Prevalence and Factors Associated with Mental Illness Symptoms among School Students Post Lockdown of the COVID-19 Pandemic in the United Arab Emirates: A Cross-Sectional National Study.

Manuscript Number: PONE-D-22-19659R1

Review Comments

1. General Comments

� Is that the prevalence of mental illness or prevalence mental illness symptoms? Why only among school students? What was unique among them since the pandemic have some sort of mental effect on the entire population? Why after the lockdown?

� Since it is self-report [self-diagnosis] what can the clinicians or the health system benefited?

� Is that common mental health illnesses or special type of mental illnesses that had occurred or anticipated to occur secondary to COVID 19?

� All the sections of the manuscript are inadequate for its scientific contents.

2. Specific Comments

� Establish the niche more and try to cite the UN and national recommendations in the background section.

� If web based, its likely that less costly. Hence, why don’t you include other segment of the population?

� How do you ensure that huma being had responded to your questions?

� What are the steps taken to ensure data quality measures, when and how?

� Have you fitted the model and its indicators were not described.

� How did you manage those with ‘mental health illnesses? Is there respondents aged less than 18 years old? Hence, how did you approach? Did they signed electronic signature?

� Is that descriptive or analytic study?

� The result should be in line with the study objective?

� The results should entail what it intends to entail in scientific paper. Similarly, address for the discussion and conclusion section. Try also to draw implications.

� Include the complete key words and avoid abbreviations without description in the abstract.

� Include International agency references E.g., WHO, IHME/GBD, UAE national health policy and strategy and COVID-19 protocol. Revisit your references.

� Where are the acknowledgement, conflict of interest, funding and consent to publish in the manuscript?

Regards,

Reviewer #3: The following study, entitled 'Prevalence and Factors Associated with Mental Illness Symptoms among School Students Post Lockdown of the COVID-19 Pandemic in the United Arab Emirates: A Cross-Sectional National Study,' investigated the prevalence of mental illness symptoms (depression, anxiety, and PTSD) among school students in the UAE after the COVID-19 pandemic lockdown. The study is relevant, but there are several points that should be considered.

1- The author needs to remove the subheading of the objective as it is part of the introduction.

2- The following sentence, in lines 101-103, is not clear: 'Parents of the surveyed students participated in the study by consenting on behalf of their under-age children and answering a series of questions within the same instrument.' Could you please clarify what the author means by 'under-age children'?

3- In method A, the paragraph (lines 158 to 161) is not clear. If the author used logistic regression to associate the status of having symptoms (nominal data; yes or no) with other student factors, it is unclear why there is a mention of the Pearson's coefficient test. Additionally, it would be better to rephrase 'Multiple linear regression' as follows: 'Multiple linear regression analyses were performed to examine the association between students' mental illness symptom scores and selected demographic and COVID-19 factors.'

4- It is not clear from the findings who the participants are: whether they are the students themselves or the parents of the students. Please provide further clarification on this matter.

5- If students responded to the questionnaire, please mention them in the table under the column 'Person Responding to this Questionnaire'.

6- The study's conclusion should not be more than one paragraph. Could you please summarize it?

Reviewer #4: I had the opportunity to thoroughly review the revised version of the manuscript, and I would like to commend the authors for their diligent efforts. Overall, the authors have made significant improvements to the paper. In the following section, I provide constructive feedback and suggestions to further enhance the quality of the manuscript.

Abstract:

1. Please indicate what PTSD is.

2. Based on Table 3, please check this sentence: "For gender differences, symptoms of the three conditions were higher in female students by 6.9%."

3. According to Table 5, the following sentence needs to be re-written correctly by either removing the values or adding three values for each condition: "Further analysis revealed that having medical problems (OR = 2.0, p < 0.001) and witnessing the death of a close family member due to COVID-19 (OR = 1.7, p < 0.001) were positive predictors associated with PTSD, depression, and anxiety."

Additionally, OR should be used instead of β.

Introduction:

1. Please add references to the sentences ending in lines 43, 53, and 55.

2. Lines 135-137 and 152 have a different font and size than the rest of the paper.

Methods:

1. I am a bit confused regarding data ownership. Do the authors of this paper own the data, or is this study a secondary data analysis of a national survey?

2. What is your response rate?

3. Based on the numbers mentioned in this paper, you have a very low response rate. Therefore, it is important to ask why the authors decided to close/end the survey. Why was the survey extended only for six months despite the low response rate?

4. What strategy was implemented to increase the response rate? Are there any other studies in the UAE with a similar response rate?

5. All these questions lead me to ask, how did you calculate your sample size?

6. Please provide more information on your pilot study. How did you assess the reliability and validity of both surveys as mentioned in the methods section?

7. It seems that your survey was administered in two languages (English and Arabic). How was the survey translated, and did you perform back translation?

Results:

1. Please add a zero in front of the decimal.

2. Report the exact p-value as it appeared in the SPSS output, even without rounding.

Discussion:

1. I don't agree with this statement, given the low response rate: "This study has adequate evidence that students in the UAE are exhibiting symptoms of mental health disorders in the post COVID-19 pandemic period, and this is consistent with recent studies across the globe."

2. The authors can't generalize their findings with this very low response rate unless they applied survey weights in their statistical analysis.

3. Since it is a national survey, I am surprised that the statistical team didn't create survey weights.

4. Please include other limitations of your study, such as being a cross-sectional study, a hypothesis generating study and the possibility of socially desirable answers and recall bias.

Conclusion:

The conclusion is too long. I suggest having a separate paragraph for the conclusion, and you might add a title for policy implications.

Reviewer #5: This study investigated the mental health symptoms and psychological distress experienced by school students in the United Arab Emirates during post lockdown of the COVID-19 pandemic. The idea was good; however, as it has been passed more than three years since the pandemic, this research can minimally be contributive to future decision-making in the respective country or region. Because the presented findings in the study have been reported in several studies before, and these kinds of findings have been well established.

However, after carefully reading the whole manuscript, I feel that this manuscript needs a major revision to achieve the merit of publication in a high-quality journal.

1. There are a few typos in the manuscript. Please go through the manuscript and correct them. E.g., in the abstract: PTDS.

2. Write the full form of PTSD in the abstract on its first appearance.

3. Rephrase the conclusion section of the abstract. It seems to me that the authors are summarizing another article. Write it in an active voice and in such a way that you performed this research.

4. At this time, there are plenty of work have been conducted on this topic, and this type of research has little implication considering the stage of covid-19. Discuss more about the justification of this study at this time and how this study is different from the previous studies conducted in the UAE on the same/similar topic. What are the limitations of the previous studies?

5. The authors are suggested to elaborate on the data collection section. The authors reported that they invited all the students in the country (nearly one million students). How do they find the number to send SMS; responding to the press release, how the participants participated in the research; what social media platforms were used and how?

6. How the authors tested the validity and reliability of the questionnaire in the studied sample? Had these instruments been validated in the previous studies?

7. Please elaborate on the scale ranges of the instruments and their cut-off values.

8. I couldn’t understand what does mean this part, “a portal for remote counseling services was communicated to all participants”?

9. Please replace the Table 2 title with a better one, “Table 2: Epidemic related information for the sample”. Epidemic could be replaced by covid-19.

10. Line 164, what does mean by ‘expat’ here?

11. Write the full form of years.

12. I am not clear about the analysis of the data. What does mean by this line, “multivariate analysis was conducted to examine the associations between mental illness symptoms and related factors by using the Pearson’s product-moment correlation coefficient test? I don’t see any such analysis in the manuscript. Were the outcome variables binary variables? If so, how were they categorized? No description in the method section. Reporting of analyses performed and the presentation of findings are quite contradictory. The authors reported multiple linear regression; however, the presentation in Table 5 and their interpretation in the result section suggest they performed a logistic regression. Again, their interpretations in the abstract are different. Reported beta coefficient. Quite unclear to me.

13. Authors are suggested to report the outcome variables among the total/overall sample also in table 3, along with the gender and age differences.

14. The discussion section is too brief. Elaborate the discussion section and make comparisons with similar studies. Suggested articles that could be helpful:

Chowdhury SR, Sunna TC, Das DC, Kabir H, Hossain A, Mahmud S, Ahmed S. Mental health symptoms among the nurses of Bangladesh during the COVID-19 pandemic. Middle East Curr Psychiatry. 2021;28(1):23. doi: 10.1186/s43045-021-00103-x.

Chowdhury SR, Kabir H, Mazumder S, Akter N, Chowdhury MR, Hossain A. Workplace violence, bullying, burnout, job satisfaction and their correlation with depression among Bangladeshi nurses: A cross-sectional survey during the COVID-19 pandemic. PLoS One. 2022 Sep 22;17(9):e0274965. doi: 10.1371/journal.pone.0274965.

Kabir H, Nasrullah SM, Hasan MK, Ahmed S, Hawlader MDH, Mitra DK. Perceived e-learning stress as an independent predictor of e-learning readiness: Results from a nationwide survey in Bangladesh. PLoS One. 2021 Oct 28;16(10):e0259281. doi: 10.1371/journal.pone.0259281.

Kabir H, Hasan MK, Mitra DK. E-learning readiness and perceived stress among the university students of Bangladesh during COVID-19: a countrywide cross-sectional study. Ann Med. 2021 Dec;53(1):2305-2314. doi: 10.1080/07853890.2021.2009908.

**********

7. PLOS authors have the option to publish the peer review history of their article (what does this mean?). If published, this will include your full peer review and any attached files.

If you choose “no”, your identity will remain anonymous but your review may still be made public.

**Do you want your identity to be public for this peer review?** For information about this choice, including consent withdrawal, please see our Privacy Policy.

Reviewer #1: No

Reviewer #3: **Yes: **Fatemeh Sharif-Askari

Reviewer #4: **Yes: **Mona Abdelrehim

Reviewer #5: No

**********

[NOTE: If reviewer comments were submitted as an attachment file, they will be attached to this email and accessible via the submission site. Please log into your account, locate the manuscript record, and check for the action link "View Attachments". If this link does not appear, there are no attachment files.]

While revising your submission, please upload your figure files to the Preflight Analysis and Conversion Engine (PACE) digital diagnostic tool, https://pacev2.apexcovantage.com/. PACE helps ensure that figures meet PLOS requirements. To use PACE, you must first register as a user. Registration is free. Then, login and navigate to the UPLOAD tab, where you will find detailed instructions on how to use the tool. If you encounter any issues or have any questions when using PACE, please email PLOS at figures@plos.org. Please note that Supporting Information files do not need this step.<quillbot-extension-portal></quillbot-extension-portal>

# Author comment: Author response to Decision Letter 1

**DOI:** 10.1371/journal.pone.0296479.r004

6 Sep 2023

Prevalence and Factors Associated with Mental Illness Symptoms among School Students Post Lockdown of the COVID-19 Pandemic in the United Arab Emirates: A Cross-Sectional National Study 

Dear Reviewers

We wish to express our gratitude to you for dedicating your time and expertise to review our work. Your meticulous evaluation and perceptive feedback have played an integral role in refining and strengthening our study. Here is the team’s response to your comments and suggestions marked in bold brown font.

Journal Requirements: 

Please review your reference list to ensure that it is complete and correct. If you have cited papers that have been retracted, please include the rationale for doing so in the manuscript text, or remove these references and replace them with relevant current references. Any changes to the reference list should be mentioned in the rebuttal letter that accompanies your revised manuscript. If you need to cite a retracted article, indicate the article’s retracted status in the References list and also include a citation and full reference for the retraction notice.

The reference list was updated. 

Additional Editor Comments (if provided):

Dear Author, reviewers still have some concerns as a mix of major and minor comments. Could you please try to address and return the manuscript with your revise version? Thank you!

All reviewers’ concerns were taken into consideration and edits were introduced accordingly. 

[Note: HTML markup is below. Please do not edit.]

Reviewers' comments:

Reviewer's Responses to Questions

Comments to the Author

1. If the authors have adequately addressed your comments raised in a previous round of review and you feel that this manuscript is now acceptable for publication, you may indicate that here to bypass the “Comments to the Author” section, enter your conflict of interest statement in the “Confidential to Editor” section, and submit your "Accept" recommendation.

Reviewer #1: All comments have been addressed

Reviewer #3: (No Response)

Reviewer #4: (No Response)

Reviewer #5: (No Response)

________________________________________

2. Is the manuscript technically sound, and do the data support the conclusions?

The manuscript must describe a technically sound piece of scientific research with data that supports the conclusions. Experiments must have been conducted rigorously, with appropriate controls, replication, and sample sizes. The conclusions must be drawn appropriately based on the data presented. 

Reviewer #1: No

Reviewer #3: Yes

Reviewer #4: Partly

Reviewer #5: Partly

________________________________________

3. Has the statistical analysis been performed appropriately and rigorously? 

Reviewer #1: No

Reviewer #3: Yes

Reviewer #4: Yes

Reviewer #5: No

________________________________________

4. Have the authors made all data underlying the findings in their manuscript fully available?

The PLOS Data policy requires authors to make all data underlying the findings described in their manuscript fully available without restriction, with rare exception (please refer to the Data Availability Statement in the manuscript PDF file). The data should be provided as part of the manuscript or its supporting information, or deposited to a public repository. For example, in addition to summary statistics, the data points behind means, medians and variance measures should be available. If there are restrictions on publicly sharing data—e.g. participant privacy or use of data from a third party—those must be specified.

Reviewer #1: Yes

Reviewer #3: Yes

Reviewer #4: Yes

Reviewer #5: Yes

________________________________________

5. Is the manuscript presented in an intelligible fashion and written in standard English?

PLOS ONE does not copyedit accepted manuscripts, so the language in submitted articles must be clear, correct, and unambiguous. Any typographical or grammatical errors should be corrected at revision, so please note any specific errors here.

Reviewer #1: Yes

Reviewer #3: No

Reviewer #4: Yes

Reviewer #5: Yes

________________________________________

6. Review Comments to the Author

Please use the space provided to explain your answers to the questions above. You may also include additional comments for the author, including concerns about dual publication, research ethics, or publication ethics. (Please upload your review as an attachment if it exceeds 20,000 characters)

Reviewer #1: Review Report

Title: Prevalence and Factors Associated with Mental Illness Symptoms among School Students Post Lockdown of the COVID-19 Pandemic in the United Arab Emirates: A Cross-Sectional National Study.

Manuscript Number: PONE-D-22-19659R1

Review Comments

1. General Comments

� Is that the prevalence of mental illness or prevalence mental illness symptoms? 

The study examined the prevalence of mental illness symptoms.

Why only among school students? What was unique among them since the pandemic have some sort of mental effect on the entire population? Why after the lockdown? 

Although the pandemic is sought to have impacted the entire population, this study focused on particularly vulnerable groups (children and adolescents) who are at crucial stages of development, both cognitively and emotionally. The pandemic-related stressors, such as isolation, online learning challenges, and fear of the virus, could have lasting effects on their mental well-being. As for why it is after lockdown, the mental health effects of the pandemic might not manifest immediately; they could have long-term implications on children's psychological well-being. By assessing mental health post-lockdown, we can capture both immediate reactions and potential delayed responses, ensuring a comprehensive understanding of the situation.

� Since it is self-report [self-diagnosis] what can the clinicians or the health system benefited?

Clinicians can potentially identify early signs of mental health concerns and implement timely interventions. This preventive approach aligns with the broader goal of promoting mental well-being from a young age and reducing the likelihood of more severe mental health issues later in life. 

� Is that common mental health illnesses or special type of mental illnesses that had occurred or anticipated to occur secondary to COVID 19?

Anxiety, depression, and post-traumatic stress disorder (PTSD) are all common mental disorders; however, the abrupt transition from lockdown to a more open society can create stress as children and adolescents adapt to changes in routines, social interactions, and expectations. The uncertainty of this transition can contribute to heightened mental challenges especially to those who directly experienced illness or loss due to COVID-19. 

� All the sections of the manuscript are inadequate for its scientific contents.

The scientific content was revisited and significant additions were introduced. 

2. Specific Comments

� Establish the niche more and try to cite the UN and national recommendations in the background section.

International perspectives and recommendations were added, please check lines 42, 44, 47, 58, 60, 69, and 73 

� If web based, its likely that less costly. Hence, why don’t you include other segment of the population?

Although authors had accessibility to the entire population, this study sought to focus on school children due to the vulnerability of this age group.

� How do you ensure that huma being had responded to your questions?

Respondents were expected to be human beings because the questionnaire link was sent out via the participating school administrations and other media means to the public.

� What are the steps taken to ensure data quality measures, when and how?

Ensuring data quality remained paramount in our study. Collaborative data handling involving multiple authors added rigor by enabling cross-validation and error minimization. Complementing this, a dependable data analysis software, IBM SPSS Statistics 26, was employed. This software not only facilitated streamlined analysis but also enhanced the consistency and reliability of our results. Additionally, our data collection was conducted using a government-powered platform (mSurvey), which provided standardized templates and guaranteed data protection and privacy, reinforcing the ethical foundation of our study.

� Have you fitted the model and its indicators were not described.

In this study, the authors had the following objectives:

1. To determine the prevalence rates of depression, anxiety, and risk for PTSD among school students in the UAE post-COVID-19 lockdown through descriptive statistics.

2. To investigate the associations between socio-demographic variables (age group, gender) and other pandemic-related factors (e.g., family member experiences) with the presence of mental health symptoms 

3. To build predictive models that identify significant predictors (e.g., age, gender, pandemic-related experiences) of depression, anxiety, and risk for PTSD among students. 

 Here is how we structured the statistical model for this data analysis:

Outcome Variables:

• Depression (binary outcome: 0 = No, 1 = Yes) 

• Anxiety (binary outcome: 0 = No, 1 = Yes) 

• Risk for PTSD (binary outcome: 0 = No, 1 = Yes)

Predictor Variables:

• Age Group (categorical: pre-adolescents, early adolescence, middle adolescence, late adolescence)

• Gender (binary: 0 = Female, 1 = Male)

• Medical Problems (binary: 0 = No, 1 = Yes)

• Close Family Member Sick/Hospitalized due to Coronavirus (binary: 0 = No, 1 = Yes)

• Close Family Member Died due to Coronavirus (binary: 0 = No, 1 = Yes)

• School Attendance (binary: 0 = Online, 1 = In-person)

Statistical Analysis Steps:

• Descriptive Statistics: We calculated frequencies and percentages for socio-demographic characteristics, pandemic-related factors, and mental health symptoms.

• Inferential analysis:

� We used Chi-squared tests (χ²) to evaluate associations between categorical variables

� P-values were computed to determine the significance of associations.

� We conducted separate logistic regression models to explore the influence of various categorical variables on the presence of symptoms (Depression, Anxiety, PTSD) generating Odds Ratios (OR), 95% Confidence Intervals (Cis), and P-values to quantify the impact of independent variables on the presence of symptoms.

� How did you manage those with ‘mental health illnesses? 

As the study did not involve the reference to medical diagnosis, all children and adolescents who responded were included. 

Is there respondents aged less than 18 years old? Hence, how did you approach? Did they signed electronic signature?

The study population included children and adolescents aged 8-18 years (Please see the method section). Invites for participation were sent out to the public via multiple corporate channels including SMS, press release, and social media platforms. In addition, school-parent communication channels served as a fundamental resource for reaching out to the study participants. As is conventional in anonymous surveys, consent was in place before starting the survey. Parents confirmed their consent in the electronic questionnaire by answering two written mandatory questions placed just at the end of the Participant Information Page. Students themselves were also requested to confirm their consent by responding to one mandatory written question just before they answered the electronic survey questions (please refer to the ethical consideration section lines 149-162). 

� Is that descriptive or analytic study?

The study incorporates both descriptive elements to paint a detailed picture of the participants and analytical elements to explore relationships and draw meaningful conclusions from the data. 

� The result should be in line with the study objective?

The study objectives were re-phrased to show the alignment (Please see lines 93-98.)

� The results should entail what it intends to entail in scientific paper. Similarly, address for the discussion and conclusion section. Try also to draw implications.

Modifications were introduced (Please check findings-line 180 and implications-line 305 sections)

� Include the complete key words and avoid abbreviations without description in the abstract.

Done

� Include International agency references E.g., WHO, IHME/GBD, UAE national health policy and strategy and COVID-19 protocol. Revisit your references.

International perspectives and recommendations were added, please check lines 42, 44, 47, 58, 60, 69, and 73. Also please check references section. 

� Where are the acknowledgement, conflict of interest, funding and consent to publish in the manuscript?

Please see the below excerpt from the “Participants Information Sheet” in the study questionnaire.

Regards,

Reviewer #3: The following study, entitled 'Prevalence and Factors Associated with Mental Illness Symptoms among School Students Post Lockdown of the COVID-19 Pandemic in the United Arab Emirates: A Cross-Sectional National Study,' investigated the prevalence of mental illness symptoms (depression, anxiety, and PTSD) among school students in the UAE after the COVID-19 pandemic lockdown. The study is relevant, but there are several points that should be considered.

1- The author needs to remove the subheading of the objective as it is part of the introduction.

Done (please see line 93)

2- The following sentence, in lines 101-103, is not clear: 'Parents of the surveyed students participated in the study by consenting on behalf of their under-age children and answering a series of questions within the same instrument.' Could you please clarify what the author means by 'under-age children'?

In this context, “under-age” means minors or children below 18 years old (per UAE Federal Law No. 3 of 2016 (Wadeema’s Law). Under-age was changed to children. 

3- In method A, the paragraph (lines 158 to 161) is not clear. If the author used logistic regression to associate the status of having symptoms (nominal data; yes or no) with other student factors, it is unclear why there is a mention of the Pearson's coefficient test. Additionally, it would be better to rephrase 'Multiple linear regression' as follows: 'Multiple linear regression analyses were performed to examine the association between students' mental illness symptom scores and selected demographic and COVID-19 factors.'

Correction done (please see the modified data analysis section) 

4- It is not clear from the findings who the participants are: whether they are the students themselves or the parents of the students. Please provide further clarification on this matter.

Parents answered parts 1 and 2 of the questionnaire, while students answered part 3. (Please see the below excerpt from the study questionnaire). 

5- If students responded to the questionnaire, please mention them in the table under the column 'Person Responding to this Questionnaire'.

Done (please see lines 181, 191, and 199)

6- The study's conclusion should not be more than one paragraph. Could you please summarize it?

Done (please revisit the conclusion section)

Reviewer #4: I had the opportunity to thoroughly review the revised version of the manuscript, and I would like to commend the authors for their diligent efforts. Overall, the authors have made significant improvements to the paper. In the following section, I provide constructive feedback and suggestions to further enhance the quality of the manuscript.

Abstract:

1. Please indicate what PTSD is.

Done (Please see line 24)

2. Based on Table 3, please check this sentence: "For gender differences, symptoms of the three conditions were higher in female students by 6.9%."

Correction done, please check line 29.

3. According to Table 5, the following sentence needs to be re-written correctly by either removing the values or adding three values for each condition: "Further analysis revealed that having medical problems (OR = 2.0, p < 0.001) and witnessing the death of a close family member due to COVID-19 (OR = 1.7, p < 0.001) were positive predictors associated with PTSD, depression, and anxiety."

Additionally, OR should be used instead of β.

Done (Please see lines 31-33)

Introduction:

1. Please add references to the sentences ending in lines 43, 53, and 55.

Done (Please see lines 39-56)

2. Lines 135-137 and 152 have a different font and size than the rest of the paper.

Done 

Methods:

1. I am a bit confused regarding data ownership. Do the authors of this paper own the data, or is this study a secondary data analysis of a national survey?

This study uses primary data collected by the study authors. 

2. What is your response rate?

In comparison to other similar studies done in the UAE, our response rate is considered relatively high. In the below mentioned UAE national studies, convenient samples of 1002, 4,426, 798 participants were included respectively. Similarly, our survey aimed to capture insights from the entire student population, which consists of a diverse and expansive group. Given the scope of our study and the logistical challenges of reaching every student across the country especially at that critical period of schools reopening, convenience and snowball sampling techniques were used. 

Abdelrahman R, Ismail ME. The psychological distress and covid-19 pandemic during lockdown: A cross-sectional study from United Arab Emirates (UAE). Heliyon. 2022;8(5). doi:10.1016/j.heliyon.2022.e09422 

Cheikh Ismail L, Mohamad MN, Bataineh MF, Ajab A, Al-Marzouqi AM, Jarrar AH, et al. Impact of the coronavirus pandemic (COVID-19) lockdown on mental health and well-being in the United Arab Emirates. Frontiers in Psychiatry. 2021;12. doi:10.3389/fpsyt.2021.633230

Vajpeyi Misra A, Mamdouh HM, Dani A, Mitchell V, Hussain HY, Ibrahim GM, et al. Impact of covid-19 pandemic on the mental health of university students in the United Arab Emirates: A cross-sectional study. BMC Psychology. 2022;10(1). doi:10.1186/s40359-022-00986-3

3. Based on the numbers mentioned in this paper, you have a very low response rate. Therefore, it is important to ask why the authors decided to close/end the survey. Why was the survey extended only for six months despite the low response rate?

While a longer survey duration could have yielded a higher response rate, the decision to open the survey for six months post COVID-19 lockdown was a careful balance between the desire for timeliness, contextual relevance, and the need to minimize potential biases introduced by a more extended period. In other words, a shorter data collection period aimed to minimize potential recall bias. As the pandemic's impact evolved, respondents' recollections of their mental health experiences could have been influenced by various external factors, thereby introducing recall bias. By keeping the survey period close to the lockdown, we aimed to capture more immediate, unaltered reflections of participants' experiences. 

4. What strategy was implemented to increase the response rate? Are there any other studies in the UAE with a similar response rate?

We employed several strategies to encourage participation. These included clear and engaging survey communications, reminders, and informative content highlighting the study's significance. As for similar studies in the UAE, here are again a list of 3 studies that used convenience and snowball sampling techniques of 1002, 4,426, 798 participants.

Abdelrahman R, Ismail ME. The psychological distress and covid-19 pandemic during lockdown: A cross-sectional study from United Arab Emirates (UAE). Heliyon. 2022;8(5). doi:10.1016/j.heliyon.2022.e09422 

Cheikh Ismail L, Mohamad MN, Bataineh MF, Ajab A, Al-Marzouqi AM, Jarrar AH, et al. Impact of the coronavirus pandemic (COVID-19) lockdown on mental health and well-being in the United Arab Emirates. Frontiers in Psychiatry. 2021;12. doi:10.3389/fpsyt.2021.633230

Vajpeyi Misra A, Mamdouh HM, Dani A, Mitchell V, Hussain HY, Ibrahim GM, et al. Impact of covid-19 pandemic on the mental health of university students in the United Arab Emirates: A cross-sectional study. BMC Psychology. 2022;10(1). doi:10.1186/s40359-022-00986-3

5. All these questions lead me to ask, how did you calculate your sample size?

A convenience and snowball sampling techniques were adopted. A data collection period was specified to be the closest to the post-lockdown period, and all participants who responded fully to the questionnaire were included. 

6. Please provide more information on your pilot study. How did you assess the reliability and validity of both surveys as mentioned in the methods section?

The questionnaire contained questions that collected biodata and 3 standardized scales (MFQ-Child self-report, SCARED-Child Version, and CRIES 8-Child Revised Impact of Events Scale). To ensure that the instrument was user-friendly and no technical difficulties were potentially existing, we ran 2 rounds of pilot testing. A group of bilingual students and parents were asked to respond anonymously to the online questionnaire and their feedback was collated to introduce modifications on the participant information sheet and the biodata questions. 

7. It seems that your survey was administered in two languages (English and Arabic). How was the survey translated, and did you perform back translation?

The questionnaire used standardized scales that are already available in both languages (Arabic and English) and tested for reliability and validity.

Children and War Foundation. Measures-Children’s Impact of Event Scale-CRIES-8 [Internet]. 2022 [cited 2023 Sept 4]. Available from: https://www.childrenandwar.org/wp-content/uploads/2019/06/Childrens-Impact-of-Event-Scale-CRIES-8-ARABIC.pdf

Tavitian L, Atwi M, Bawab S, Hariz N, Zeinoun P, Khani M, et al. The Arabic mood and feelings questionnaire: Psychometrics and validity in a clinical sample. Child Psychiatry & Human Development. 2013;45(3):361–8. doi:10.1007/s10578-013-0406-6

Hariz N, Bawab S, Atwi M, Tavitian L, Zeinoun P, Khani M, et al. Reliability and validity of the Arabic screen for child anxiety related emotional disorders (scared) in a clinical sample. Psychiatry Research. 2013;209(2):222–8. doi:10.1016/j.psychres.2012.12.002 

Results:

1. Please add a zero in front of the decimal.

Done

2. Report the exact p-value as it appeared in the SPSS output, even without rounding.

Done

Discussion:

1. I don't agree with this statement, given the low response rate: "This study has adequate evidence that students in the UAE are exhibiting symptoms of mental health disorders in the post COVID-19 pandemic period, and this is consistent with recent studies across the globe."

While response rate is a consideration, our study's approach, coupled with global trends, substantiates the assertion of mental health symptoms among UAE students post COVID-19. However, we appreciate your concern and in this revised manuscript, we will transparently discuss limitations tied to the response rate and their potential implications on generalizability (line 290). Here is a supportive global study:

Xiong, J., Lipsitz, O., Nasri, F., Lui, L. M. W., Gill, H., Phan, L., ... & McIntyre, R. S. (2020). Impact of COVID-19 pandemic on mental health in the general population: A systematic review. Journal of affective disorders, 277, 55-64.

2. The authors can't generalize their findings with this very low response rate unless they applied survey weights in their statistical analysis.

While survey weights were not applied in our analysis, we believe that the impact on our results is limited. Our study's comprehensive population-based approach aimed to capture a diverse cross-section of students in the UAE, encompassing a wide range of socio-demographic characteristics. As such, we are confident that our findings provide meaningful insights into the mental health landscape among students during the post-COVID-19 pandemic period. We value your input and will ensure to transparently discuss this aspect in our manuscript to provide a well-rounded understanding of our research (Please see lines 287-290).

3. Since it is a national survey, I am surprised that the statistical team didn't create survey weights.

While applying survey weights would have been no doubt helpful, none of the similar studies conducted in the UAE mentioned that survey weights were applied despite them using a similar range of participants. However, this perspective is addressed in the limitations section. 

4. Please include other limitations of your study, such as being a cross-sectional study, a hypothesis generating study and the possibility of socially desirable answers and recall bias.

Other limitations are added (Please see lines 284-290)

Conclusion:

The conclusion is too long. I suggest having a separate paragraph for the conclusion, and you might add a title for policy implications.

Conclusion is rewritten.

Reviewer #5: This study investigated the mental health symptoms and psychological distress experienced by school students in the United Arab Emirates during post lockdown of the COVID-19 pandemic. The idea was good; however, as it has been passed more than three years since the pandemic, this research can minimally be contributive to future decision-making in the respective country or region. Because the presented findings in the study have been reported in several studies before, and these kinds of findings have been well established.

However, after carefully reading the whole manuscript, I feel that this manuscript needs a major revision to achieve the merit of publication in a high-quality journal.

1. There are a few typos in the manuscript. Please go through the manuscript and correct them. E.g., in the abstract: PTDS.

Edits done

2. Write the full form of PTSD in the abstract on its first appearance.

Done

3. Rephrase the conclusion section of the abstract. It seems to me that the authors are summarizing another article. Write it in an active voice and in such a way that you performed this research.

Done

4. At this time, there are plenty of work have been conducted on this topic, and this type of research has little implication considering the stage of covid-19. Discuss more about the justification of this study at this time and how this study is different from the previous studies conducted in the UAE on the same/similar topic. What are the limitations of the previous studies?

Kindly note that the study review process was delayed by PLOS One for a full year. Despite the inconvenience this slow process has caused to authors, we are still confident that the study holds its unique contributions, particularly given the absence of similar studies conducted in the UAE. Moreover, in this second review, we have incorporated additional relevant references to further enrich the study's context.

5. The authors are suggested to elaborate on the data collection section. The authors reported that they invited all the students in the country (nearly one million students). How do they find the number to send SMS; responding to the press release, how the participants participated in the research; what social media platforms were used and how?

For participant recruitment, we adopted a comprehensive strategy. In the initial phase, we engaged potential participants through school and parental communication avenues. Furthermore, we harnessed the electronic communication platforms affiliated with the authors' corporate connections. This entailed disseminating the invitation and study link to the public across official websites, press releases, and various social media platforms such as Facebook, Instagram, and Twitter. For SMS communication, we utilized the established networks of corporate facilities, including hospitals and clinics, to circulate the invitation to the employees’ families who met the inclusion criteria. This creative dissemination method facilitated broader access to our study's invitation.

6. How the authors tested the validity and reliability of the questionnaire in the studied sample? Had these instruments been validated in the previous studies?

All used scales are standardized (Mood and Feelings Questionnaire (MFQ)-Child self-report, Screen for Child Anxiety Related Disorders (SCARED)-Child Version, and Child Revised Impact of Events Scale (CRIES). 

7. Please elaborate on the scale ranges of the instruments and their cut-off values.

Done. Please check lines 198-204

8. I couldn’t understand what does mean this part, “a portal for remote counseling services was communicated to all participants”?

A professional mental health support hotline was made accessible for participants in case they needed relevant support (Please see lines 161-162). Please check the below excerpt from the study questionnaire.

9. Please replace the Table 2 title with a better one, “Table 2: Epidemic related information for the sample”. Epidemic could be replaced by covid-19.

Done

10. Line 164, what does mean by ‘expat’ here?

In this context, expat means non-Emirati nationals.

11. Write the full form of years.

Modifications done 

12. I am not clear about the analysis of the data. What does mean by this line, “multivariate analysis was conducted to examine the associations between mental illness symptoms and related factors by using the Pearson’s product-moment correlation coefficient test? I don’t see any such analysis in the manuscript. Were the outcome variables binary variables? If so, how were they categorized? No description in the method section. Reporting of analyses performed and the presentation of findings are quite contradictory. The authors reported multiple linear regression; however, the presentation in Table 5 and their interpretation in the result section suggest they performed a logistic regression. Again, their interpretations in the abstract are different. Reported beta coefficient. Quite unclear to me.

Data analysis section is re-written for clarification.

13. Authors are suggested to report the outcome variables among the total/overall sample also in table 3, along with the gender and age differences.

Done (Please check lines 198-204)

14. The discussion section is too brief. Elaborate the discussion section and make comparisons with similar studies. Suggested articles that could be helpful.

Thank you for providing these references. We have carefully reviewed the discussion sections in the references you suggested and made relevant edits to the corresponding section in our study accordingly. 

Chowdhury SR, Sunna TC, Das DC, Kabir H, Hossain A, Mahmud S, Ahmed S. Mental health symptoms among the nurses of Bangladesh during the COVID-19 pandemic. Middle East Curr Psychiatry. 2021;28(1):23. doi: 10.1186/s43045-021-00103-x.

Chowdhury SR, Kabir H, Mazumder S, Akter N, Chowdhury MR, Hossain A. Workplace violence, bullying, burnout, job satisfaction and their correlation with depression among Bangladeshi nurses: A cross-sectional survey during the COVID-19 pandemic. PLoS One. 2022 Sep 22;17(9):e0274965. doi: 10.1371/journal.pone.0274965.

Kabir H, Nasrullah SM, Hasan MK, Ahmed S, Hawlader MDH, Mitra DK. Perceived e-learning stress as an independent predictor of e-learning readiness: Results from a nationwide survey in Bangladesh. PLoS One. 2021 Oct 28;16(10):e0259281. doi: 10.1371/journal.pone.0259281.

Kabir H, Hasan MK, Mitra DK. E-learning readiness and perceived stress among the university students of Bangladesh during COVID-19: a countrywide cross-sectional study. Ann Med. 2021 Dec;53(1):2305-2314. doi: 10.1080/07853890.2021.2009908.

________________________________________

7. PLOS authors have the option to publish the peer review history of their article (what does this mean?). If published, this will include your full peer review and any attached files.

If you choose “no”, your identity will remain anonymous but your review may still be made public.

Do you want your identity to be public for this peer review? For information about this choice, including consent withdrawal, please see our Privacy Policy.

Reviewer #1: No

Reviewer #3: Yes: Fatemeh Sharif-Askari

Reviewer #4: Yes: Mona Abdelrehim

Reviewer #5: No

Attachment Submitted filename: Response to Reviewers (29-08-2023).docx Click here for additional data file.

# Peer review: Decision Letter 2

**DOI:** 10.1371/journal.pone.0296479.r005

**Authors:** Humayun Kabir

2 Oct 2023

PONE-D-22-19659R2Prevalence and Factors Associated with Mental Illness Symptoms among School Students Post Lockdown of the COVID-19 Pandemic in the United Arab Emirates: A Cross-Sectional National StudyPLOS ONE

Dear Dr. Ghader,

Thank you for submitting your manuscript to PLOS ONE. After careful consideration, we feel that it has merit but does not fully meet PLOS ONE’s publication criteria as it currently stands. Therefore, we invite you to submit a revised version of the manuscript that addresses the points raised during the review process.

Please submit your revised manuscript by Nov 16 2023 11:59PM. If you will need more time than this to complete your revisions, please reply to this message or contact the journal office at plosone@plos.org. When you're ready to submit your revision, log on to https://www.editorialmanager.com/pone/ and select the 'Submissions Needing Revision' folder to locate your manuscript file.

Please include the following items when submitting your revised manuscript:

- A rebuttal letter that responds to each point raised by the academic editor and reviewer(s). You should upload this letter as a separate file labeled 'Response to Reviewers'.
- A marked-up copy of your manuscript that highlights changes made to the original version. You should upload this as a separate file labeled 'Revised Manuscript with Track Changes'.
- An unmarked version of your revised paper without tracked changes. You should upload this as a separate file labeled 'Manuscript'.

If you would like to make changes to your financial disclosure, please include your updated statement in your cover letter. Guidelines for resubmitting your figure files are available below the reviewer comments at the end of this letter.

If applicable, we recommend that you deposit your laboratory protocols in protocols.io to enhance the reproducibility of your results. Protocols.io assigns your protocol its own identifier (DOI) so that it can be cited independently in the future. For instructions see: https://journals.plos.org/plosone/s/submission-guidelines#loc-laboratory-protocols. Additionally, PLOS ONE offers an option for publishing peer-reviewed Lab Protocol articles, which describe protocols hosted on protocols.io. Read more information on sharing protocols at https://plos.org/protocols?utm_medium=editorial-email&utm_source=authorletters&utm_campaign=protocols.

We look forward to receiving your revised manuscript.

Kind regards,

Humayun Kabir

Academic Editor

PLOS ONE

Journal Requirements:

Please review your reference list to ensure that it is complete and correct. If you have cited papers that have been retracted, please include the rationale for doing so in the manuscript text, or remove these references and replace them with relevant current references. Any changes to the reference list should be mentioned in the rebuttal letter that accompanies your revised manuscript. If you need to cite a retracted article, indicate the article’s retracted status in the References list and also include a citation and full reference for the retraction notice.

[Note: HTML markup is below. Please do not edit.]

Reviewers' comments:

Reviewer's Responses to Questions

**Comments to the Author**

1. If the authors have adequately addressed your comments raised in a previous round of review and you feel that this manuscript is now acceptable for publication, you may indicate that here to bypass the “Comments to the Author” section, enter your conflict of interest statement in the “Confidential to Editor” section, and submit your "Accept" recommendation.

Reviewer #1: All comments have been addressed

Reviewer #4: All comments have been addressed

Reviewer #5: (No Response)

**********

2. Is the manuscript technically sound, and do the data support the conclusions?

The manuscript must describe a technically sound piece of scientific research with data that supports the conclusions. Experiments must have been conducted rigorously, with appropriate controls, replication, and sample sizes. The conclusions must be drawn appropriately based on the data presented. 

Reviewer #1: Partly

Reviewer #4: Yes

Reviewer #5: Yes

**********

3. Has the statistical analysis been performed appropriately and rigorously? 

Reviewer #1: Yes

Reviewer #4: Yes

Reviewer #5: Yes

**********

4. Have the authors made all data underlying the findings in their manuscript fully available?

The PLOS Data policy requires authors to make all data underlying the findings described in their manuscript fully available without restriction, with rare exception (please refer to the Data Availability Statement in the manuscript PDF file). The data should be provided as part of the manuscript or its supporting information, or deposited to a public repository. For example, in addition to summary statistics, the data points behind means, medians and variance measures should be available. If there are restrictions on publicly sharing data—e.g. participant privacy or use of data from a third party—those must be specified.

Reviewer #1: Yes

Reviewer #4: Yes

Reviewer #5: Yes

**********

5. Is the manuscript presented in an intelligible fashion and written in standard English?

PLOS ONE does not copyedit accepted manuscripts, so the language in submitted articles must be clear, correct, and unambiguous. Any typographical or grammatical errors should be corrected at revision, so please note any specific errors here.

Reviewer #1: Yes

Reviewer #4: Yes

Reviewer #5: Yes

**********

6. Review Comments to the Author

Please use the space provided to explain your answers to the questions above. You may also include additional comments for the author, including concerns about dual publication, research ethics, or publication ethics. (Please upload your review as an attachment if it exceeds 20,000 characters)

Reviewer #1: The manuscript still fails to ;

1.have more strong backgroind for its update and rationale

2. Assemble the fragments of ideas

3.Clarity of the sub sections

4. Grammer and language flows

5. Singluar Vs plural e.g. method

6. Update the references

7. The tables and figures should be self explanatory

8. Flawed use of cut off points e.g. p value less than 0.05

9.Ensureb the completeness of the sub sections on details

Regards,

Reviewer #4: I would like to thank the authors for addressing the reviewers' comments.

The following are my comments:

1. Although the authors mentioned using a convenient snowball sampling technique, they still need to determine the minimum number of respondents required to conduct multiple regression analyses.

Please refer to the following paper:

https://doi.org/10.1207/s15327906mbr2603_7

Don't worry; you already have enough participants.

2. In Table 3, please report the exact p-value

Reviewer #5: Thank you for addressing most of the comments. I have a few minor edit suggestion:

1. Abstract, "For gender differences, symptoms of the three conditions were higher in female students (9.2%) compared to male peers (7.7%) (p<0.05)." Three conditions were higher in the female, but only one % is reported. This percentage represents which condition?

2. "similarly, witnessing the death of a close family member due to COVID-19 (OR=1.7, p<0.01) were positive predictors associated with PTDS, depression, and anxiety." This OR represents which conditions?

3. In the statistical analysis section, the authors are suggested to report how the variables were selected for multivariable logistics regression model.

4. In my earlier revision, I suggested the authors to expand the discussion section discussing all the significant variables and also suggested a few articles for their help. However, the authors did not elaborate the discussion section.

Thanks

**********

7. PLOS authors have the option to publish the peer review history of their article (what does this mean?). If published, this will include your full peer review and any attached files.

If you choose “no”, your identity will remain anonymous but your review may still be made public.

**Do you want your identity to be public for this peer review?** For information about this choice, including consent withdrawal, please see our Privacy Policy.

Reviewer #1: No

Reviewer #4: **Yes: **Mona Abdelrehim

Reviewer #5: No

**********

[NOTE: If reviewer comments were submitted as an attachment file, they will be attached to this email and accessible via the submission site. Please log into your account, locate the manuscript record, and check for the action link "View Attachments". If this link does not appear, there are no attachment files.]

While revising your submission, please upload your figure files to the Preflight Analysis and Conversion Engine (PACE) digital diagnostic tool, https://pacev2.apexcovantage.com/. PACE helps ensure that figures meet PLOS requirements. To use PACE, you must first register as a user. Registration is free. Then, login and navigate to the UPLOAD tab, where you will find detailed instructions on how to use the tool. If you encounter any issues or have any questions when using PACE, please email PLOS at figures@plos.org. Please note that Supporting Information files do not need this step.

# Author comment: Author response to Decision Letter 2

**DOI:** 10.1371/journal.pone.0296479.r006

31 Oct 2023

Dear Reviewers,

Thank you again for your valuable feedback. Please find below the modifications we introduced based on your comments.

Regards,

________________________________________

Reviewer #1: The manuscript still fails to;

1.have stronger background for its update and rationale

The introduction section was revisited and additional details were introduced.

2. Assemble the fragments of ideas

We have diligently worked on assembling fragmented ideas to create a cohesive and logical flow into the introduction of the manuscript. We ensured a smooth transition between sections and ideas, improving the overall readability and comprehension of the content.

3.Clarity of the sub sections

We acknowledge the need for enhancing clarity in sub-sections. We have revised and organized sub-sections in the method section to provide a clear and coherent structure, ensuring that each subsection has a distinct focus and effectively contributes to the narrative of the manuscript.

4. Grammar and language flows

We carefully reviewed and edited the manuscript to refine the grammar and language flow. Attention was given to sentence structure, word choice, and coherence to ensure a clear and engaging presentation of the research.

5. Singular Vs plural e.g. method

We have meticulously reviewed and ensured the consistent usage of singular or plural forms, as appropriate, throughout the manuscript, paying close attention to maintaining clarity and precision in conveying our intended meaning. 

6. Update the references

In the revised version of the manuscript, we have meticulously cross-verified all references to ensure they are current and relevant to the subject matter. The inclusion of older references in this study is primarily tied to the use of well-established evidence-based questionnaires, which have stood the test of time in terms of reliability, validity, and acceptance within the academic and professional communities. These questionnaires, designed to measure specific constructs or variables, have become gold standards in their respective domains and have been extensively validated over the years.

7. The tables and figures should be self-explanatory

We fully agree with the importance of making our tables and figures self-explanatory to enhance the clarity and comprehensibility of the research presented. In our revised manuscript, we have taken the following measures to address this concern:

Improved Captions and Detailed Legends:

We have ensured that each table is accompanied by a clear and informative caption that succinctly explains the content and purpose. The captions is crafted to provide sufficient context for readers to interpret the data and findings without necessarily referring to the main text.

8. Flawed use of cut off points e.g. p value less than 0.05

SPSS, like other statistical software, typically reports p-values to three decimal places. However, it is important to note that this level of precision does not imply a higher level of significance. P-values below 0.05 are often reported as "< 0.001" or "< .001" for practical reasons related to formatting and readability. The Exact p- values in table 3 are added.

9.Ensure the completeness of the sub sections on details

We have carefully reviewed and enhanced the completeness of all subsections to ensure that they contain adequate and comprehensive information, providing readers with a thorough understanding of the content.

Regards,

Reviewer #4: I would like to thank the authors for addressing the reviewers' comments.

The following are my comments:

1. Although the authors mentioned using a convenient snowball sampling technique, they still need to determine the minimum number of respondents required to conduct multiple regression analyses.

For snowball sampling, the sample size is often not pre-determined with a specific formula. Instead, the sample size grows organically as new participants are referred by the initial participants.

Please refer to the following paper:

 Age of student (years) Gender of student

 Pre-adolescence (<10 years)

[n=1285] Early adolescence (10-13 years)

[n=1403] Middle adolescence (14-17 years)

[n=888] Late adolescence (18 years)

[n=67] 

p-value 

Male

[n=1895] 

Female

[n=1800] 

p-value

Symptoms of one condition 

 Depression only 218 (17.0) 246 (17.5) 153 (17.2) 18 (26.9) 0.22 301 (15.9) 333 (18.5) <0.05

 Anxiety only 288 (22.4) 328 (23.4) 219 (24.7) 16 (23.9) 0.68 377 (19.9) 482 (26.8) <0.01

 PTSD only 499 (38.8) 593 (42.3) 371 (41.8) 27 (40.3) 0.30 730 (38.5) 765 (42.5) <0.05

Symptoms of two conditions 

 Depression and anxiety 125 (9.7) 153 (10.9) 114 (12.8) 12 (17.9) <0.05

177 (9.3) 224 (12.4) <0.01

 Depression and PTSD 146 (11.4) 184 (13.1) 104 (11.7) 11 (16.4) 0.35 207 (10.9) 237 (13.2) <0.05

 Anxiety and PTSD 198 (15.4) 254 (18.1) 151 (17.0) 12 (17.9) 0.31 279 (14.7) 340 (18.9) <0.01

Symptoms of three conditions 

 Depression, anxiety, and PTSD 96 (7.5) 130 (9.3) 81 (9.1) 8 (11.9) 0.24 146 (7.7) 166 (9.2) 0.09

https://doi.org/10.1207/s15327906mbr2603_7

Don't worry; you already have enough participants.

We understand the importance of establishing an adequate sample size, especially when conducting multiple regression analyses, to ensure statistical power and reliability in our findings. We have taken this into account in our study and carefully considered the need for an appropriate number of respondents to support our analyses.

Regarding the provided reference, we have reviewed the paper and its recommendations regarding the determination of the minimum sample size for regression analyses. We agree that having sufficient participants is essential for the robustness of our analysis.

We assure you that we have carefully considered the statistical requirements and have taken steps to ensure that our study has an appropriate number of participants to support our multiple regression analyses and allow us to draw meaningful conclusions from the data.

2. In Table 3, please report the exact p-value

Standard Reporting of P-Values:

SPSS, like other statistical software, typically reports p-values to three decimal places. However, it is important to note that this level of precision does not imply a higher level of significance. P-values below 0.05 are often reported as "< 0.001" or "< .001" for practical reasons related to formatting and readability.

Reviewer #5: Thank you for addressing most of the comments. I have a few minor edit suggestion:

1. Abstract, "For gender differences, symptoms of the three conditions were higher in female students (9.2%) compared to male peers (7.7%) (p<0.05)." Three conditions were higher in the female, but only one % is reported. This percentage represents which condition?

Done! For gender differences, symptoms of the three conditions (Depression, anxiety, and PTSD) were higher in female students (9.2%) compared to male peers (7.7%) (p<0.05).

2. "similarly, witnessing the death of a close family member due to COVID-19 (OR=1.7, p<0.01) were positive predictors associated with PTDS, depression, and anxiety." This OR represents which conditions?

Done! The OR represents all three conditions (PTSD, depression, and anxiety)

3. In the statistical analysis section, the authors are suggested to report how the variables were selected for multivariable logistics regression model.

In the variable selection process for the multivariable logistic regression model, we carefully considered several factors to ensure a robust and appropriate selection of variables that are relevant to our research question. These considerations were based on both statistical and substantive reasoning.

Relevance to Research Question:

We assessed the theoretical relevance of each variable to the research question and objectives of our study. Variables directly related to the phenomenon under investigation were given higher priority.

Literature Review:

A comprehensive literature review guided the selection of variables. We incorporated findings from previous research and existing theoretical frameworks to identify variables with established associations with the outcomes of interest.

Clinical Expertise:

We consulted with domain experts, including clinicians and subject matter experts, to ensure that the variables selected align with clinical knowledge and expertise related to the research topic.

Statistical Significance:

Statistical significance of variables was evaluated using appropriate tests, such as chi-square tests o, depending on the nature of the variables (categorical or continuous). Variables with a significant association (p-value below a pre-defined threshold, e.g., p < 0.05) were considered for inclusion.

4. In my earlier revision, I suggested the authors to expand the discussion section discussing all the significant variables and also suggested a few articles for their help. However, the authors did not elaborate the discussion section.

We have added the suggested articles in the discussion section: 

Chowdhury SR, Sunna TC, Das DC, Kabir H, Hossain A, Mahmud S, Ahmed S. Mental health symptoms among the nurses of Bangladesh during the COVID-19 pandemic. Middle East Curr Psychiatry. 2021;28(1):23. doi: 10.1186/s43045-021-00103-x.

Chowdhury SR, Kabir H, Mazumder S, Akter N, Chowdhury MR, Hossain A. Workplace violence, bullying, burnout, job satisfaction and their correlation with depression among Bangladeshi nurses: A cross-sectional survey during the COVID-19 pandemic. PLoS One. 2022 Sep 22;17(9):e0274965. doi: 10.1371/journal.pone.0274965.

Attachment Submitted filename: Response to reviewers(31-10-2023).docx Click here for additional data file.

# Peer review: Decision Letter 3

**DOI:** 10.1371/journal.pone.0296479.r007

**Authors:** Humayun Kabir

19 Nov 2023

PONE-D-22-19659R3Prevalence and Factors Associated with Mental Illness Symptoms among School Students Post Lockdown of the COVID-19 Pandemic in the United Arab Emirates: A Cross-Sectional National StudyPLOS ONE

Dear Dr. Ghader,

Thank you for submitting your manuscript to PLOS ONE. After careful consideration, we feel that it has merit but does not fully meet PLOS ONE’s publication criteria as it currently stands. Therefore, we invite you to submit a revised version of the manuscript that addresses the points raised during the review process.

Please submit your revised manuscript by December 19, 2023. If you will need more time than this to complete your revisions, please reply to this message or contact the journal office at plosone@plos.org. When you're ready to submit your revision, log on to https://www.editorialmanager.com/pone/ and select the 'Submissions Needing Revision' folder to locate your manuscript file.

Please include the following items when submitting your revised manuscript:

- A rebuttal letter that responds to each point raised by the academic editor and reviewer(s). You should upload this letter as a separate file labeled 'Response to Reviewers'.
- A marked-up copy of your manuscript that highlights changes made to the original version. You should upload this as a separate file labeled 'Revised Manuscript with Track Changes'.
- An unmarked version of your revised paper without tracked changes. You should upload this as a separate file labeled 'Manuscript'.

If you would like to make changes to your financial disclosure, please include your updated statement in your cover letter. Guidelines for resubmitting your figure files are available below the reviewer comments at the end of this letter.

If applicable, we recommend that you deposit your laboratory protocols in protocols.io to enhance the reproducibility of your results. Protocols.io assigns your protocol its own identifier (DOI) so that it can be cited independently in the future. For instructions see: https://journals.plos.org/plosone/s/submission-guidelines#loc-laboratory-protocols. Additionally, PLOS ONE offers an option for publishing peer-reviewed Lab Protocol articles, which describe protocols hosted on protocols.io. Read more information on sharing protocols at https://plos.org/protocols?utm_medium=editorial-email&utm_source=authorletters&utm_campaign=protocols.

We look forward to receiving your revised manuscript.

Kind regards,

Humayun Kabir

Academic Editor

PLOS ONE

Journal Requirements:

Please review your reference list to ensure that it is complete and correct. If you have cited papers that have been retracted, please include the rationale for doing so in the manuscript text, or remove these references and replace them with relevant current references. Any changes to the reference list should be mentioned in the rebuttal letter that accompanies your revised manuscript. If you need to cite a retracted article, indicate the article’s retracted status in the References list and also include a citation and full reference for the retraction notice.

[Note: HTML markup is below. Please do not edit.]

Reviewers' comments:

Reviewer's Responses to Questions

**Comments to the Author**

1. If the authors have adequately addressed your comments raised in a previous round of review and you feel that this manuscript is now acceptable for publication, you may indicate that here to bypass the “Comments to the Author” section, enter your conflict of interest statement in the “Confidential to Editor” section, and submit your "Accept" recommendation.

Reviewer #1: All comments have been addressed

Reviewer #4: All comments have been addressed

Reviewer #5: (No Response)

**********

2. Is the manuscript technically sound, and do the data support the conclusions?

The manuscript must describe a technically sound piece of scientific research with data that supports the conclusions. Experiments must have been conducted rigorously, with appropriate controls, replication, and sample sizes. The conclusions must be drawn appropriately based on the data presented. 

Reviewer #1: Partly

Reviewer #4: Yes

Reviewer #5: Partly

**********

3. Has the statistical analysis been performed appropriately and rigorously? 

Reviewer #1: Yes

Reviewer #4: Yes

Reviewer #5: (No Response)

**********

4. Have the authors made all data underlying the findings in their manuscript fully available?

The PLOS Data policy requires authors to make all data underlying the findings described in their manuscript fully available without restriction, with rare exception (please refer to the Data Availability Statement in the manuscript PDF file). The data should be provided as part of the manuscript or its supporting information, or deposited to a public repository. For example, in addition to summary statistics, the data points behind means, medians and variance measures should be available. If there are restrictions on publicly sharing data—e.g. participant privacy or use of data from a third party—those must be specified.

Reviewer #1: Yes

Reviewer #4: (No Response)

Reviewer #5: (No Response)

**********

5. Is the manuscript presented in an intelligible fashion and written in standard English?

PLOS ONE does not copyedit accepted manuscripts, so the language in submitted articles must be clear, correct, and unambiguous. Any typographical or grammatical errors should be corrected at revision, so please note any specific errors here.

Reviewer #1: Yes

Reviewer #4: Yes

Reviewer #5: (No Response)

**********

6. Review Comments to the Author

Please use the space provided to explain your answers to the questions above. You may also include additional comments for the author, including concerns about dual publication, research ethics, or publication ethics. (Please upload your review as an attachment if it exceeds 20,000 characters)

Reviewer #1: We thank the authors for addressing our comments in tge previous version and the following are our concern in this version ;

1. It fails to present whether descriptive or analytic study and the rationale behind

2.use of present tense e.g 'are' in the abstract section .

3.covinience and snow ball sampling for quantitative study is unusual.

4.The background fails to cite the global and national initiatives promised to prevent and control mental health and its symptoms

5.The data quality assurance is missed e.g is all the collected data complete ?

6. Ethics issues:

_Age of respondents and the way consent was secured

_What was done for those with mental health illness

_Is there funding?If so the role of funder should be stated.

_Authors contributions is not described

7.The tables are many /5/.Present some of the tables in narration form and it lacks self explanation. In addition usw figurea for most attracting findings.

8. The discussion is not in line with the guidelines of the journal.

9 Statistics

-The percentage are not exactly 100%

-Use confidence interval while presenting percentage

Regards,

Reviewer #4: Thank you for addressing the previous comments; the manuscript is ready for publication.

On a side note, it is essential to emphasize that reporting the exact P values is crucial in research. It is essential to note that not all p-values below 0.05 should be reported as <0.001, however, when the software output displayed the p-value as "000," the researchers reported it as <0.001.

Reviewer #5: Thanks for addressing most of the comments. However, the manuscript still requires improvement to get it published in a high-quality journal.

A few comments below:

1. Discuss elaborately the tools used for assessing three psychological conditions. Items of the tools, their cut-off values, and the cut-off used to determine the presence of three conditions. The scales' reliability and validity in this population.

2. Discuss in the statistical analysis section how the variables were selected for multivariable analysis.

3. Avoid using the informal short form in the result section (eg. yrs).

4. The presentation in the table is messy and difficult to understand. Improve the presentation of the data in the tables. You can make the cell borders visible to make the table clear.

Thanks.

**********

7. PLOS authors have the option to publish the peer review history of their article (what does this mean?). If published, this will include your full peer review and any attached files.

If you choose “no”, your identity will remain anonymous but your review may still be made public.

**Do you want your identity to be public for this peer review?** For information about this choice, including consent withdrawal, please see our Privacy Policy.

Reviewer #1: No

Reviewer #4: **Yes: **Mona Abdelrehim

Reviewer #5: No

**********

[NOTE: If reviewer comments were submitted as an attachment file, they will be attached to this email and accessible via the submission site. Please log into your account, locate the manuscript record, and check for the action link "View Attachments". If this link does not appear, there are no attachment files.]

While revising your submission, please upload your figure files to the Preflight Analysis and Conversion Engine (PACE) digital diagnostic tool, https://pacev2.apexcovantage.com/. PACE helps ensure that figures meet PLOS requirements. To use PACE, you must first register as a user. Registration is free. Then, login and navigate to the UPLOAD tab, where you will find detailed instructions on how to use the tool. If you encounter any issues or have any questions when using PACE, please email PLOS at figures@plos.org. Please note that Supporting Information files do not need this step.

# Author comment: Author response to Decision Letter 3

**DOI:** 10.1371/journal.pone.0296479.r008

23 Nov 2023

Dear Reviewers,

Thank you again for your valuable feedback. Please find below the modifications we introduced based on your comments.

Regards,

________________________________________

6. Review Comments to the Author

Reviewer #1: We thank the authors for addressing our comments in tge previous version and the following are our concern in this version ;

1. It fails to present whether descriptive or analytic study and the rationale behind

The study uses the descriptive study design (please check lines 136-142).

Rationale: 

Literature affirms that descriptive studies are best used to describe the distribution of one or more variables, devoid of causal links. Furthermore, this type of studies is considered instrumental in measuring the prevalence of a disease or of a risk factor in a population (Aggarawal & Ranganathan, 2019). In this study, the focus is on discerning the prevalence and factors associated with mental illness symptoms among school students in the COVID-19 post-lockdown phase. Recognizing the efficacy of descriptive studies in unveiling patterns and trends, researchers anticipate that this design will uncover commonalities, variations, or trends among students, offering invaluable insights for targeted interventions in the realm of mental health symptoms.

Aggarwal R, Ranganathan P. Study designs: Part 2 - Descriptive studies. Perspect Clin Res. 2019 Jan-Mar;10(1):34-36. doi: 10.4103/picr.PICR_154_18. PMID: 30834206; PMCID: PMC6371702.

2.use of present tense e.g 'are' in the abstract section .

Done (please check lines no. 35-36)

3.covinience and snow ball sampling for quantitative study is unusual.

Due to the limitations imposed by the COVID-19 pandemic, many researchers on similar studies (please see references listed below) used the convenience and snowball sampling techniques (Abdelrahman, 2022; Cheikh, 2021; Vajpeyi, 2022). More precisely, this technique was adopted by researchers in the UAE due to the inherent interconnectedness of the population under study. Leveraging existing networks and connections within the target population facilitated access to individuals who may have otherwise been challenging to reach through traditional sampling methods. We acknowledge the limitations associated with convenience and snowball sampling and we have provided a thorough discussion of these limitations in the revised manuscript.

References:

1. Abdelrahman R, Ismail ME. The psychological distress and covid-19 pandemic during lockdown: A cross-sectional study from United Arab Emirates (UAE). Heliyon. 2022;8(5). doi:10.1016/j.heliyon.2022.e09422 

2. Cheikh Ismail L, Mohamad MN, Bataineh MF, Ajab A, Al-Marzouqi AM, Jarrar AH, et al. Impact of the coronavirus pandemic (COVID-19) lockdown on mental health and well-being in the United Arab Emirates. Frontiers in Psychiatry. 2021;12. doi:10.3389/fpsyt.2021.633230

3. Vajpeyi Misra A, Mamdouh HM, Dani A, Mitchell V, Hussain HY, Ibrahim GM, et al. Impact of covid-19 pandemic on the mental health of university students in the United Arab Emirates: A cross-sectional study. BMC Psychology. 2022;10(1). doi:10.1186/s40359-022-00986-3

4.The background fails to cite the global and national initiatives promised to prevent and control mental health and its symptoms

More global and national initiatives were added (please check lines 80-87).

5.The data quality assurance is missed e.g is all the collected data complete ?

Data quality assurance was added (please check lines 190-203).

6. Ethics issues:

_Age of respondents and the way consent was secured

Age of respondents ranged from 8-18 years (please check lines 148-149).

Students were requested to confirm their consent by responding to one mandatory written question just before they answered the electronic survey questions. Participants were informed they could stop completing the questionnaire or refrain from submitting their responses if they felt uncomfortable at any stage during completion. Participation in the study was voluntary. Participants had the full right to withdraw at any time without the need to justify their actions. (Please check lines 239-243)

_What was done for those with mental health illness:

Considering that students with special needs were excluded from the study, all students who met the inclusion criteria were included in data analysis. 

_Is there funding?If so the role of funder should be stated.

The study did not receive any funding. However, the publication fees will be covered by the Emirates Health Services which is recognized in the acknowledgement section.

_Authors contributions is not described

Authors’ contributions followed the universal requirement for authorship as well as those of PLOS ONE. 

https://hms.harvard.edu/sites/default/files/assets/Sites/Ombuds/files/AUTHORSHIP%20GUIDELINES.pdf

7.The tables are many /5/.Present some of the tables in narration form and it lacks self explanation. In addition usw figurea for most attracting findings.

To address your concern about self-explanation, we enhanced the narrative descriptions accompanying the tables to ensure a comprehensive and clear understanding of the presented data. Furthermore, we have added one figure.

As for the number of tables, we believe all of them are useful for presenting the multiple datasets in an organized manner. 

8. The discussion is not in line with the guidelines of the journal.

We again revisited the guidelines of the journal as shown below. One suggestion may be to merge the discussion and conclusion parts under one section labeled “Discussion”. Perhaps the editor can guide us in this regard. 

https://journals.plos.org/plosone/s/submission-guidelines

9 Statistics

-The percentage are not exactly 100%

-Use confidence interval while presenting percentage

We would like to clarify that the discrepancy in percentages is attributed to inapplicable/unanswered responses in the dataset. Some participants chose not to respond to certain items, resulting in a non-valid dataset for certain variables. If you add these responses together it equals 100%. We acknowledge the importance of transparency in reporting and will explicitly mention in the revised manuscript that the percentages presented are based on the number of valid responses for each item. 

We appreciate your suggestion regarding the use of confidence intervals to present percentages in our study. While we acknowledge the utility of confidence intervals in many statistical contexts, it is important to note that presenting confidence intervals for percentages is not a common practice in certain fields, particularly in reporting prevalence. Percentages are typically reported as point estimates, representing the proportion of a sample that exhibits a particular characteristic or response. Confidence intervals are more commonly used for continuous variables or when estimating parameters of a population distribution. We have already presented confidence intervals in Table 5. Applying confidence intervals to percentages may lead to misinterpretations, as they are inherently bounded between 0% and 100%, and confidence intervals could extend beyond these limits. To enhance clarity and adherence to standard reporting practices in our field, we have chosen to present percentages as point estimates in the manuscript.

Reviewer #4: Thank you for addressing the previous comments; the manuscript is ready for publication.

On a side note, it is essential to emphasize that reporting the exact P values is crucial in research. It is essential to note that not all p-values below 0.05 should be reported as <0.001, however, when the software output displayed the p-value as "000," the researchers reported it as <0.001.

Thank you for the side note, we have already reported the exact p values in the last revision. We appreciate your attention to detail, particularly regarding the reporting of P values. We acknowledge the importance of accurate and transparent reporting in research. There were specific instances where the software output displayed a P-value as "<0.001,"and so we reported the same in the revised version and we understand the necessity of accurately representing such values rather than automatically reporting them.

Reviewer #5: Thanks for addressing most of the comments. However, the manuscript still requires improvement to get it published in a high-quality journal.

A few comments below:

1. Discuss elaborately the tools used for assessing three psychological conditions. Items of the tools, their cut-off values, and the cut-off used to determine the presence of three conditions. The scales' reliability and validity in this population.

Tools are elaborated on page number 7.

Cut off is mentioned on page 14

2. Discuss in the statistical analysis section how the variables were selected for multivariable analysis.

we have enhanced the statistical analysis section to provide a detailed discussion on the criteria and rationale behind the selection of variables for the multivariable analysis. This included a thorough explanation of the factors considered, such as variable significance, relevance to the research question, and pre-established criteria for inclusion. By addressing this aspect more explicitly, we aimed at offering readers a clearer understanding of the methodological choices made during the analysis. 

3. Avoid using the informal short form in the result section (eg. yrs).

Done

4. The presentation in the table is messy and difficult to understand. Improve the presentation of the data in the tables. You can make the cell borders visible to make the table clear.

The formatting of the table is done as requested.

Thanks.

________________________________________

Attachment Submitted filename: Response to reviewers(Revised-Nov23-2023).docx Click here for additional data file.

# Peer review: Decision Letter 4

**DOI:** 10.1371/journal.pone.0296479.r009

**Authors:** Humayun Kabir

14 Dec 2023

Prevalence and Factors Associated with Mental Illness Symptoms among School Students Post Lockdown of the COVID-19 Pandemic in the United Arab Emirates: A Cross-Sectional National Study

PONE-D-22-19659R4

Dear Dr. Ghader,

We’re pleased to inform you that your manuscript has been judged scientifically suitable for publication and will be formally accepted for publication once it meets all outstanding technical requirements.

Within one week, you’ll receive an e-mail detailing the required amendments. When these have been addressed, you’ll receive a formal acceptance letter and your manuscript will be scheduled for publication.

An invoice for payment will follow shortly after the formal acceptance. To ensure an efficient process, please log into Editorial Manager at http://www.editorialmanager.com/pone/, click the 'Update My Information' link at the top of the page, and double check that your user information is up-to-date. If you have any billing related questions, please contact our Author Billing department directly at authorbilling@plos.org.

If your institution or institutions have a press office, please notify them about your upcoming paper to help maximize its impact. If they’ll be preparing press materials, please inform our press team as soon as possible -- no later than 48 hours after receiving the formal acceptance. Your manuscript will remain under strict press embargo until 2 pm Eastern Time on the date of publication. For more information, please contact onepress@plos.org.

Kind regards,

Humayun Kabir

Academic Editor

PLOS ONE

Additional Editor Comments (optional):

The current version of the manuscript is ready for publishing. There is no need for additional modification. 

Reviewers' comments:

Reviewer's Responses to Questions

**Comments to the Author**

1. If the authors have adequately addressed your comments raised in a previous round of review and you feel that this manuscript is now acceptable for publication, you may indicate that here to bypass the “Comments to the Author” section, enter your conflict of interest statement in the “Confidential to Editor” section, and submit your "Accept" recommendation.

Reviewer #1: All comments have been addressed

Reviewer #5: All comments have been addressed

**********

2. Is the manuscript technically sound, and do the data support the conclusions?

The manuscript must describe a technically sound piece of scientific research with data that supports the conclusions. Experiments must have been conducted rigorously, with appropriate controls, replication, and sample sizes. The conclusions must be drawn appropriately based on the data presented. 

Reviewer #1: Partly

Reviewer #5: Yes

**********

3. Has the statistical analysis been performed appropriately and rigorously? 

Reviewer #1: No

Reviewer #5: (No Response)

**********

4. Have the authors made all data underlying the findings in their manuscript fully available?

The PLOS Data policy requires authors to make all data underlying the findings described in their manuscript fully available without restriction, with rare exception (please refer to the Data Availability Statement in the manuscript PDF file). The data should be provided as part of the manuscript or its supporting information, or deposited to a public repository. For example, in addition to summary statistics, the data points behind means, medians and variance measures should be available. If there are restrictions on publicly sharing data—e.g. participant privacy or use of data from a third party—those must be specified.

Reviewer #1: Yes

Reviewer #5: (No Response)

**********

5. Is the manuscript presented in an intelligible fashion and written in standard English?

PLOS ONE does not copyedit accepted manuscripts, so the language in submitted articles must be clear, correct, and unambiguous. Any typographical or grammatical errors should be corrected at revision, so please note any specific errors here.

Reviewer #1: Yes

Reviewer #5: Yes

**********

6. Review Comments to the Author

Please use the space provided to explain your answers to the questions above. You may also include additional comments for the author, including concerns about dual publication, research ethics, or publication ethics. (Please upload your review as an attachment if it exceeds 20,000 characters)

Reviewer #1: We thank the authors for addressing some of our comments and the following two issues atmre not addresswd;

1. In ethics . I ma asking what was done after they have PTDS?

2. It is a mist to present the confidence interval.

And the following are my commnets in this version ;

A. The abstract lacks clarity.

B.Reframe the title for the purpose of the study .

C.The background is insufficient for its contents.

D.The methods order, content and merging of some of the sub section is needed.

E. Tool and how the tool was introduced in UAE should be described well.

F. The resukts and the discussion as well as the consequent sections are entirely flawed.

G. Revisit language and grammar issues e.g aged 8 to 18 can be rewritten.as aged 8 to 18 years.

Regards,

Reviewer #5: (No Response)

**********

7. PLOS authors have the option to publish the peer review history of their article (what does this mean?). If published, this will include your full peer review and any attached files.

If you choose “no”, your identity will remain anonymous but your review may still be made public.

**Do you want your identity to be public for this peer review?** For information about this choice, including consent withdrawal, please see our Privacy Policy.

Reviewer #1: No

Reviewer #5: **Yes: **Saifur Rahman Chowdhury

**********

# Editor report: Acceptance letter

**DOI:** 10.1371/journal.pone.0296479.r010

**Authors:** Humayun Kabir

23 Jan 2024

PONE-D-22-19659R4 

PLOS ONE

Dear Dr. Ghader, 

I'm pleased to inform you that your manuscript has been deemed suitable for publication in PLOS ONE. Congratulations! Your manuscript is now being handed over to our production team.

At this stage, our production department will prepare your paper for publication. This includes ensuring the following:

* All references, tables, and figures are properly cited

* All relevant supporting information is included in the manuscript submission,

* There are no issues that prevent the paper from being properly typeset

If revisions are needed, the production department will contact you directly to resolve them. If no revisions are needed, you will receive an email when the publication date has been set. At this time, we do not offer pre-publication proofs to authors during production of the accepted work. Please keep in mind that we are working through a large volume of accepted articles, so please give us a few weeks to review your paper and let you know the next and final steps. 

Lastly, if your institution or institutions have a press office, please let them know about your upcoming paper now to help maximize its impact. If they'll be preparing press materials, please inform our press team within the next 48 hours. Your manuscript will remain under strict press embargo until 2 pm Eastern Time on the date of publication. For more information, please contact onepress@plos.org.

If we can help with anything else, please email us at customercare@plos.org.

Thank you for submitting your work to PLOS ONE and supporting open access. 

Kind regards, 

PLOS ONE Editorial Office Staff

on behalf of

Dr. Humayun Kabir 

Academic Editor

PLOS ONE
